# The evolution of consultation practices with general practitioners and nephrologists for patients with chronic kidney disease before and after the COVID-19 pandemic in France

**DOI:** 10.1007/s40620-025-02376-5

**Published:** 2025-07-27

**Authors:** Juliette Piveteau, Sahar Bayat, Cécile Vigneau, Cécile Couchoud, Maxime Raffray

**Affiliations:** 1 EHESP, CNRS, Inserm, Arènes-UMR 6051, RSMS, U1309, Univ Rennes, Rennes, France; 2 Univ Rennes, CHU Rennes, INSERM, EHESP, IRSET (Institut de Recherche en Santé, Environnement et Travail)-UMR S 1085, Rennes, France; 3 Renal Epidemiology and Information Network (REIN) Registry, Biomedicine Agency, Saint-Denis-La-Plaine, France; 4 Division of Clinical Epidemiology, Department of Medicine Solna, Karolinska Institutet, Solna, Sweden

**Keywords:** COVID-19, Chronic kidney disease, Healthcare utilization, Teleconsultation

## Abstract

**Background:**

The COVID-19 pandemic led to concerns about disruptions in the follow-up of chronic diseases, including chronic kidney disease (CKD). Here, we assessed the COVID-19 pandemic impact on healthcare use by patients with CKD in France.

**Methods:**

We used the French National Health Data System (SDNS) that contains data on outpatient and inpatient healthcare of the whole French population. Using a validated algorithm, we identified two CKD cohorts based on their healthcare utilization: (i) the 2019 cohort (pandemic-exposed) and (ii) the 2017 cohort (comparator). We followed these cohorts for 2 years and compared consultations (in-person and teleconsultation) with a general practitioner (GP) and a nephrologist and all-cause hospitalizations (excluding COVID-19 as primary diagnosis). We stratified comparisons by age group and sex.

**Results:**

We identified 4,866,096 individuals with CKD in 2017 and 5,089,706 in 2019. During the first year of follow-up, 95.2% and 6.4% of patients in the 2017 cohort had at least one consultation with a GP and with a nephrologist, respectively, *versus* 94% and 6.3% in the 2019 cohort. Teleconsultations compensated for the reduction of in-person GP and nephrologist consultations throughout the lockdown periods in 2020 and 2021 (40.5% of patients in the 2019 cohort had at least one in-person consultation and 52.5% an in-person or tele-consultation with a GP). Hospitalizations ≥ 24 h decreased in 2020 and 2021 (-10%).

**Conclusions:**

In France, outpatient care for CKD was maintained during the 2 years following the COVID-19 pandemic, thanks to teleconsultations. The persistently lower inpatient care utilization warrants further investigation.

**Graphical abstract:**

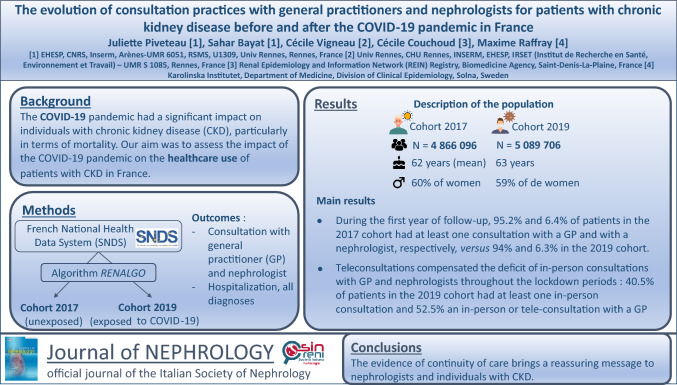

**Supplementary Information:**

The online version contains supplementary material available at 10.1007/s40620-025-02376-5.

## Introduction

The coronavirus disease 2019 (COVID-19) led to the death of more than 7 million individuals worldwide as of September 2024 [1]. On March 12, 2020, after the confirmation of 118,000 cases of COVID-19 in 114 countries, including 4,291 deaths, the World Health Organization defined COVID-19 as a pandemic [2]. Besides the direct casualties, the pandemic greatly disrupted the functioning of many aspects of society, including healthcare service delivery [3]. Direct healthcare disruptions were reported early in the pandemic, such as reallocation of resources and discontinuation of emergency and acute care [4]. Additionally, countries deployed a range of policies to control the pandemic, including lockdowns [5]. This resulted in a global decrease in healthcare use [6], leading to concerns about its potential long-term negative consequences on the population health. Indeed, healthcare disruptions have been associated with subsequent preventable hospital admissions [7] and delays in the diagnosis and treatment of several non-communicable diseases, including cardiovascular diseases [8] and cancer [9].

Chronic Kidney Disease (CKD) is a silent disease that slowly progresses over months or years, with a prevalence of 10% in the adult population [10]. This disease is defined as “the presence of markers of kidney damage or a decrease in the estimated glomerular filtration rate below 60 ml/min/1.73 m^2^ for more than three months, regardless of its cause” [11]. Early detection and close follow-up with adherence to care guidelines are crucial for better outcomes, including preventing or better anticipating kidney failure [12, 13]. In France, CKD patients should be co-managed by GP, nephrologist, and nurse practitioners, with follow-up frequency increasing as kidney function declines. Guidelines range from yearly GP visits at early stages to monthly visits alternating nephrologist, nurse practitioner and GP at stage 5 non-treated by kidney replacement therapy (KRT) [13].

Individuals with CKD are particularly vulnerable to COVID-19, with increased mortality [14–18]. Few studies have investigated the changes in healthcare use and potential disruptions specifically in individuals with non-dialysis-dependent CKD. A US study found a reduction in overall healthcare use during the early months of the pandemic followed by an incomplete rebound [19]. However, data are scare in countries with universal healthcare coverage. Our aim was to assess the impact of the COVID-19 pandemic on the healthcare use of patients with CKD in France.

## Materials and methods

### Data sources

This observational retrospective study was carried out using data from the French National Health Data System (SNDS). The SNDS contains all inpatient and outpatient claims and hospital discharges for 99.5% of the 67 million inhabitants of France (i.e., beneficiaries of the universal healthcare coverage system). The data include information on reimbursed drug deliveries and inpatient/outpatient medical procedures and diagnoses. Some sociodemographic information on the beneficiaries is also available, including age, sex and area of residence [20].

### Study population

The study population was identified in the SNDS data for 2017 and for 2019 using the validated algorithm *RENALGO* [21]. This algorithm identifies individuals with CKD using combinations of diagnoses codes, drug prescriptions and procedures found in the SNDS. Because the SNDS does not contain lab values, we could not distinguish individuals based on their CKD stage. This yielded two cohorts of individuals with CKD who were followed for two years. The 2019 cohort included individuals exposed to the COVID 19 pandemic (follow-up from January 1, 2020 to December 31, 2021). The 2017 cohort was considered the control population and included individuals unexposed to the pandemic (follow-up from January 1, 2018 to December 31, 2019). Patients who initiated KRT (dialysis or kidney transplant) or who died during the year of identification (from January 1 to December 31, 2017 for the 2017 cohort and from January 1 to December 31, 2019 for the 2019 cohort) were excluded.

### Data collection

From the SNDS, the following data were collected at baseline (i.e., 2017 or 2019): age, sex, region of residence and city-level deprivation index (in quintiles: Q5 is the most disadvantaged [22]). Additionally, data on comorbidities were collected including diabetes, cancer, cardiovascular diseases, psychiatric diseases, neurological or degenerative diseases, chronic respiratory diseases, inflammatory or rare diseases, HIV or AIDS, vascular risk management (without pathology), and psychotropic treatments (without pathology). The complete French social security methodology for comorbidity identification is available at https://www.assurance-maladie.ameli.fr/sites/default/files/2024_methode-reperage-pathologies_cartographie.pdf. During the 2 years of follow-up of each cohort, all outpatient visits carried out by a general practitioner (GP) or nephrologist were extracted from the SNDS. Consultations were divided into teleconsultations and in-person consultations using the specific French Nomenclature of Care Reimbursement (*Nature des prestations*: 1191, 1192, 1157, 1164, 1096, 1097, 1193, 1194, 1158, 1165, 1172, 1174). All hospitalizations were also extracted and divided into day hospital care (< 24 h of stay, all diagnoses) and hospitalizations for > 24 h (all diagnoses). Hospitalizations with COVID-19 as the main or related diagnosis (ICD-10 codes: U07.10 COVID-19, respiratory form, virus identified; U07.11 COVID-19, respiratory form, virus unidentified; U07.14 COVID-19, other clinical forms, virus identified; and U07.15 COVID-19, other clinical forms, virus unidentified) were excluded.

### Statistical analysis

We assessed the comparability of the two cohorts. Their characteristics were reported using frequencies and percentages for categorical variables and median and interquartile range for continuous variables. The two years of follow-up after the year of identification (N) were divided into eight periods according to the lockdown periods in France during the pandemic: period 1 (pre-pandemic, 01/01/*N* + 1–16/03/*N* + 1), period 2 (first lockdown, 17/03/*N* + 1–10/05/*N* + 1), period 3 (post-first lockdown, 11/05/*N* + 1–29/10/*N* + 1), period 4 (second lockdown, 30/10/*N* + 1–14/12/*N* + 1), period 5 (post-second lockdown, 15/12*N* + 1–02/04/*N* + 2), period 6 (third lockdown, 03/04/*N* + 2–02/05/*N* + 2), period 7 (post-third lockdown, 03/05/*N* + 2–09/06/*N* + 2) and period 8 (post-pandemic, 10/06/*N* + 2–31/12/*N* + 2) (Supplementary Figure S1).

Mortality rates and cumulative death incidence were compared between cohorts, stratified by age groups (< 60, 60–74 and ≥ 75 years of age). The percentage of patients who had at least one healthcare service utilization in each period (first and second year of follow-up) was calculated. For each period, the sum of person-time alive was calculated and used as the denominator in order to neutralize the effect of death. These percentages were compared in the two cohorts for all periods and annually and for the total population, stratified by age (same groups as above), and between sexes (men and women). Because we included a nationwide population and because the two cohorts were not mutually exclusive, we chose to not perform statistical tests of difference and report p-values. The cumulative incidence of individuals receiving the first consultation during the follow-up was calculated and plotted for both cohorts to assess the trend of outpatient care use (GP and nephrologist). Additional exploratory analyses were carried out by stratifying the sample according to age groups and sex, region of residence and city-level deprivation index.

## Results

### Description of the study population

Using the *RENALGO* algorithm, 4,866,096 individuals with CKD were identified in 2017 and 5,089,706 in 2019 (total *n* = 1,890,460 patients) (Supplementary Figure S2). The two cohorts had similar characteristics and comorbidity profiles. The mean age was 62 years (standard deviation = 20.6) in the 2017 cohort and 63 years (standard deviation = 20.4) in the 2019 cohort. In both cohorts, women represented 59% of patients and 25% of patients had diabetes (Supplementary Table S1).

### Comparison of mortality rates

At the end of the 2 years of follow-up, 9.1% and 9.7% of patients had died in the 2017 cohort and in the 2019 cohort, respectively. The mortality rate was higher in the 2019 cohort than 2017 cohort during periods 2, 4 and 6 (i.e. the lockdown periods) (Supplementary Figure S3). In the 2019 cohort, the COVID-19 mortality rate was higher during the lockdown periods (19.8% and 21.2% during the first and the second lockdown) than during the post-pandemic period (4.0%).

### Comparison of outpatient healthcare utilization

**Fig. 1:**
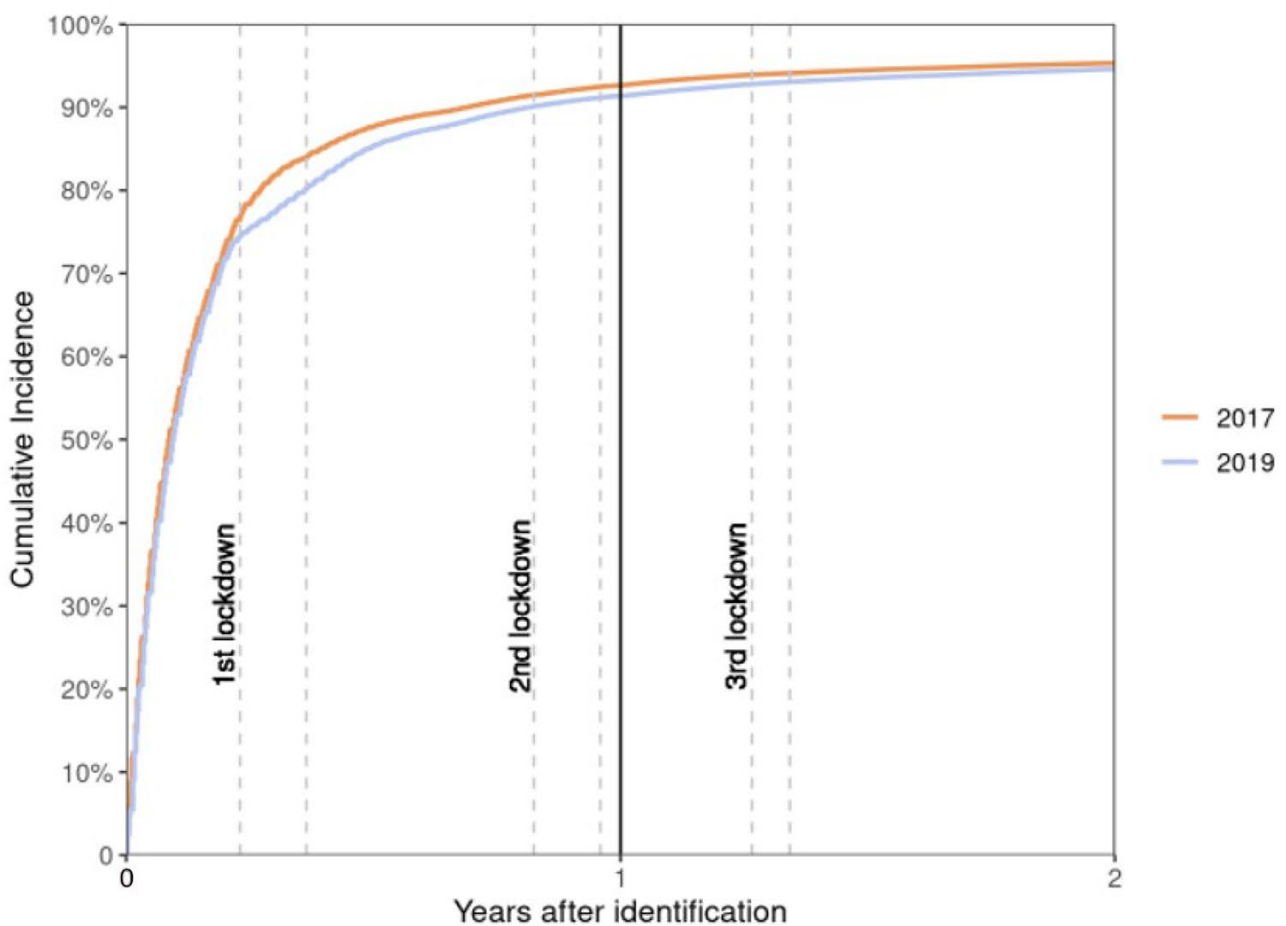
Cumulative incidence of a first GP consultation in the 2017 and 2019 cohorts during the follow-up

In the 2019 cohort, 94% of the 5,089,706 patients had at least one consultation (in-person or teleconsultation) with a GP during the first year of follow-up (*N* + 1). This was slightly lower (− 1.3%) than in the 2017 cohort (95.2%) (Table 1). The percentage was similar between cohorts during the second year of follow-up (N + 2). The greatest decreases in the 2019 cohort were observed during period 2: − 12.9% when considering all consultations with a GP (60.3% of patients in the 2017 cohort had at least one consultation vs 52.7% in the 2019 cohort) and -32.8% when considering only in-person consultations. Comparison of the cumulative incidence of the first consultation with a GP during the follow-up period showed that despite an apparent reduction during period 2 (first lockdown), the percentage of patients in cohort 2019 with at least one consultation steadily increased over the 2 years of follow-up (Fig. 1).

**Table 1:** Percentages of patients in each cohort who consulted a GP at least once and a nephrologist at least once in each period in person-time, adjusted for deaths

		All consultations (in-person consultations and teleconsultations^a^)						Only in-person consultations						
		% of patients with ≥ 1 GP consultation			% of patients with ≥ 1 nephrologist consultation			% of patients with ≥ 1 GP consultation			% of patients with ≥ 1 nephrologist consultation			
		2017 cohort	2019 cohort	Relative difference^b^	2017 cohort	2019 cohort	Relative difference^b^	2017 cohort	2019 cohort	Relative difference^b^		2017 cohort	2019 cohort	Relative difference^b^
Year *N* + 1		95.2	94.0	− 1.3	6.4	6.3	− 1.6	95.2	94.0	− 1.3		6.4	6.3	− 1.6
Year *N* + 2		92.5	92.4	− 0.1	6.1	6.5	6.6	92.4	92.4	0		6.1	6.4	4.9
Period 1	01/01/*N* + 1–16/03/*N* + 1	73.9	72.4	− 2.2	2.4	2.5	4.2	73.9	72.3	− 2.2		2.4	2.4	0
Period 2	17/03/*N* + 1–10/05/*N* + 1	60.3	52.5	− 12.9	1.6	0.8	− 50	60.3	40.5	− 32.8		1.6	0.4	− 75
Period 3	11/05/*N* + 1–29/10/*N* + 1	86.4	93.5	8.2	4.2	4.3	2.4	86.4	84.3	− 2.4		4.2	4.0	− 4.8
Period 4	30/10/*N* + 1–14/12/*N* + 1	56.8	55.6	− 2.1	1.7	1.5	− 11.8	56.8	51.2	− 9.9		1.6	1.4	− 12.5
Period 5	15/12/*N* + 1–02/04/*N* + 2	79.1	83.9	6.1	2.9	3.1	6.9	79.0	76.5	− 3.2		2.9	2.9	0
Period 6	03/04/*N* + 2–02/05/*N* + 2	38.9	38.7	− 0.5	0.9	0.9	0	38.9	36.6	− 5.9		0.9	0.8	− 11.1
Period 7	03/05/*N* + 2–09/06/*N* + 2	46.7	47.3	1.3	1.3	1.3	0	46.7	45.2	− 3.2		1.2	1.3	8.3
Period 8	10/06/*N* + 2–31/12/*N* + 2	85.2	90.7	6.5	4.5	4.7	4.4	85.1	85.2	0.1		4.5	4.7	4.4

^a^No teleconsultation was found in 2018 because it was only introduced as a procedure in France in late 2018

^b^(2017 proportion − 2019 proportion)/2017 proportion

**Fig. 2:**
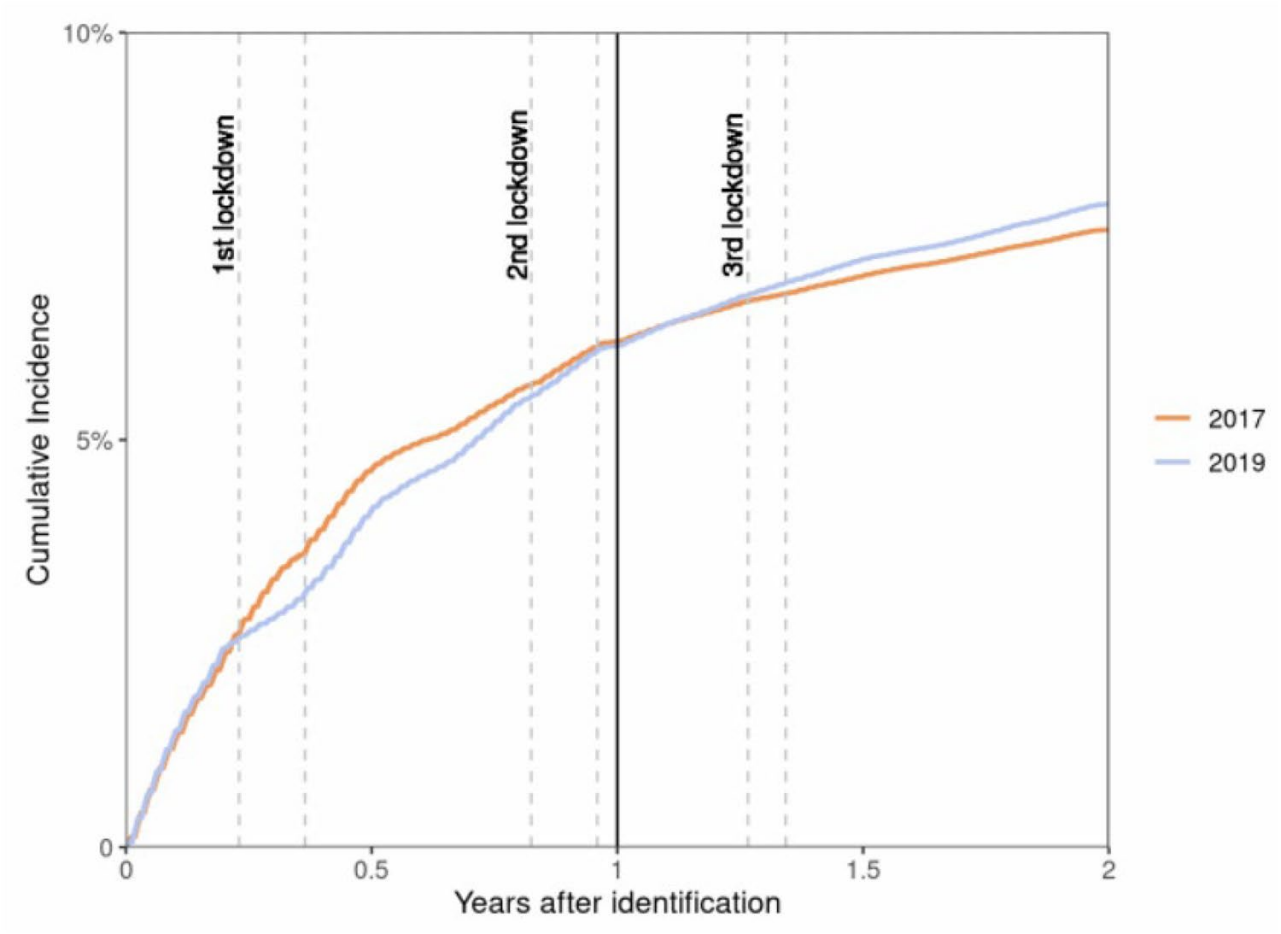
Cumulative incidence of a first nephrologist consultation in the 2017 and 2019 cohorts during the follow-up

Overall, there was no difference in the proportion of patients who had at least one consultation (in-person or teleconsultation) with a nephrologist during the first year of follow-up (6.4% in the 2017 cohort vs 6.3% in the 2019 cohort). Regarding GP consultations, in the 2019 cohort, the proportion of patients who had at least one consultation with a nephrologist was lower during period 2, even when considering both teleconsultations and in-person consultations (0.8% vs 1.6% in the 2017 cohort, 50% decrease). Comparison of the cumulative incidence of the first consultation with a nephrologist during the follow-up period again showed a reduction in period 2 followed by a progressive increase over time (Fig. 2). When only in-person consultations were considered, the proportion of patients with at least one consultation with a nephrologist strongly decreased in the 2019 cohort during periods 2, 4 and 6 (i.e., first, second and third lockdown) by 75%, 12.5% and 11.1% compared with the 2017 cohort.

### Comparison of inpatient healthcare utilization

Table 2 presents the percentages of patients with at least one < 24 h hospitalization or one ≥ 24 h hospitalization during the follow-up. The percentage of patients with at least one < 24 h hospitalization slightly decreased in the 2019 cohort during the first year of follow-up (27.3% in the 2017 cohort vs 26% in the 2019 cohort). The most significant decrease (-36.5%) was observed during period 2. However, during the second year of follow-up, slightly more patients in the 2019 cohort had at least one < 24 h hospitalization (25% in 2017 cohort vs 26.2% in the 2019 cohort).

**Table 2:** Percentage of patients in each cohort with at least one hospital stay in each period, in person-time, adjusted for deaths

		% of patients with ≥ 1 hospital stay for < 24 h			% of patients with ≥ 1 hospital stay > 24 h		
		2017 cohort	2019 cohort	Relative difference	2017 cohort	2019 cohort	Relative difference
Year *N* + 1		27.3	26	− 4.8	27.0	24.1	− 10.7
Year *N* + 2		25.0	26.2	4.8	21.2	19.1	− 9.9
Period 1	01/01/*N* + 1–16/03/*N* + 1	10.4	11.1	6.7	10.1	9.7	− 4.0
Period 2	17/03/*N* + 1–10/05/*N* + 1	7.4	4.7	− 36.5	6.3	4.3	− 31.7
Period 3	11/05/*N* + 1–29/10/*N* + 1	15.1	15.5	2.6	12.8	11.7	− 8.6
Period 4	30/10/*N* + 1–14/12/*N* + 1	6.3	6.2	− 1.6	4.3	3.3	− 23.3
Period 5	15/12/*N* + 1–02/04/*N* + 2	10.8	11.4	5.6	8.8	7.3	− 17.0
Period 6	03/04/*N* + 2–02/05/*N* + 2	4.3	4.4	2.3	2.7	2.2	− 18.5
Period 7	03/05/*N* + 2–09/06/*N* + 2	5.1	5.6	9.8	3.3	3.0	− 9.1
Period 8	10/06/*N* + 2–31/12/*N* + 2	15.8	17.0	7.6	12.9	11.9	− 7.8

The percentage of patients with at least one ≥ 24 h hospitalization was lower in the 2019 cohort during the first year of follow-up (27% in the 2017 cohort vs 24.1% in the 2019 cohort) and also in the second year of follow-up and throughout all periods. The most important reductions in the 2019 cohort were observed during the lockdown periods (31.7%, -23.3% and -18.5% for periods 2, 4 and 6, respectively).

### Stratification analyses

The additional analyses did not show any difference in outpatient consultation rates between cohorts when stratified according to sex (Supplementary Figure S4), age groups (Supplementary Figure S5), and by sex and age groups (Supplementary Table S2). Similar results were obtained when patients were stratified based on the city-level deprivation index (Supplementary Table S3), despite the higher consultation rates in socioeconomically advantaged areas and higher proportions of hospital use in the most deprived areas. The cumulative incidence of GP and nephrologist consultations followed different dynamics between regions (Supplementary Figures S6, S7). However, there was no difference in the proportion of patients with a consultation at the end of the 2 years of follow-up between the 2017 and 2019 cohort.

## Discussion

In this nationwide population-based study, we assessed the impact of the COVID-19 pandemic on healthcare use by patients with CKD in France. The mortality rate was higher in the 2019 cohort during the lockdown periods than in the 2017 cohort in the equivalent periods. Indeed, the COVID-19 mortality rates were higher during the lockdown periods and declined slowly after the third lockdown. The level of outpatient healthcare use remained stable, with minimal changes in consultation rates with GPs and nephrologists. Thanks to teleconsultations, the proportion of patients who had at least one consultation did not change much between cohorts. The consistency of these results across age groups, sex and socioeconomic indicators provides reassurance that outpatient healthcare continuity was largely maintained despite the pandemic. Nonetheless, the persistent decline in inpatient healthcare use raises concerns about possible implications for long-term health outcomes in individuals with CKD.

Our findings are consistent with previous French reports. Tuppin et al. found only a -3% decrease of the proportion of the total French population with at least one consultation with a GP in 2020 compared with 2019 [6]. In addition, our study showed that this proportion remained stable for individuals with CKD exposed to the COVID-19 pandemic, both in 2020 and 2021, despite a greater decrease at specific time points during the pandemic (i.e. the three lockdown periods). It also found that the proportions of individuals with CKD having a consultation with a nephrologist remained the same at the yearly level.

One important finding of our study is that teleconsultations allowed compensating the deficit of in-person consultations with GPs and nephrologists throughout the lockdown periods in 2020 and 2021. A study in the US reported a decrease of outpatient consultations for individuals with CKD (*N* = 250,000) that was not sufficiently compensated by teleconsultations in 2020 and 2021 [23]. This difference can be attributed to variations in healthcare coverage as well as methodological considerations, since healthcare use was calculated differently (mean number of consultations per individual in the US study vs percentage of individuals with at least one consultation in our study). It is worth noting that in France, the definition of a teleconsultation for reimbursement eligibility was extended at the time of the first lockdown from video consultation to telephone call. This could have influenced the frequencies, thus making international comparisons difficult. Nevertheless, teleconsultations (by telephone or videoconference) have shown their benefits for patient and healthcare continuity in primary practice and nephrology settings [24, 25]. It seems to truly have replaced in-patient consultations during the lockdowns because there was no increase in consultations after the lockdown. Indeed, teleconsultation was probably a good option for well-known patients with chronic diseases, but more difficult for patients with acute medical problems, as reported by Tuppin et al. [20].

Hospitalization for ≥ 24 h of individuals with CKD persistently decreased in the 2019 cohort during the two years of followup, and particularly during the lockdown periods. Several explanations can be hypothesized. First, the 2021 inpatient activity in France was still lower than the 2019 pre-pandemic activity [26], with cancellation and postponement of hospitalizations. Mounting evidence suggests that this could be of concern for long-term health outcomes [7–9, 27]. Moreover, lockdowns and related restrictions might have reduced the environmental triggers to hospitalizations. Similarly, Mesnier et al. observed in France a decrease in hospital admissions for acute myocardial infarctions following the first lockdown [28]. Therefore, the reasons for hospital admissions should be better explored to understand the persistent decrease.

By focusing on the population of individuals with CKD not treated by KRT, our study completes previous reports on the impact of the COVID-19 pandemic on dialysis initiation and kidney transplantation. The Renal Epidemiology and Information Network (REIN) registry has prospectively collected new cases of KRT in France since 2002 [29]. Based on their numbers, the incidence of individuals treated by KRT decreased between 2019 and 2022 (175.6 per million inhabitants in 2019, 164.7 in 2020, 168.6 in 2021 and 163 in 2022). One explanation for this decrease is the death of individuals with advanced stages of CKD who died due to COVID-19 before reaching the need for KRT (https://www.agence-biomedecine.fr/Les-chiffres-du-R-E-I-N). Similar data were reported in the US [30]. Additionally, disruptions in care could have negatively impacted the progression to kidney failure, particularly for patients with advanced stages of CKD. Indeed, those patients require a close follow-up in nephrology and potential preparation to KRT. The REIN registry prospectively records whether dialysis treatment is initiated in emergency or not, and the REIN numbers indicate a sustained slight increase of emergency initiations (in 2019: 25% of hemodialysis initiations, 2020: 26%, 2021: 29%, 2022: 30%) [31, 32]. In the initial phase of the pandemic in 2020, premature deaths of individuals with CKD due to COVID-19 might explain why the proportion of emergency initiations did not change. The sustained increase in later years could indicate a residual, delayed effect of changes in care delivery (i.e. less preparation to dialysis), which strengthen the future importance of studying the quality of care. Kidney transplantation activity was paused for two months before progressively resuming in 2021 and beyond. By 2024, the number of kidney transplants returned to 2019 levels (3757).

In early 2020, France introduced a new capitation payment model [33]. Providers that opt-in receive an annual payment per patient enrolled (CKD stage 4 and stage 5 non-treated by dialysis). This new policy could be a confounder to our analysis. First, receiving the payment relies on collecting the number of visits with a nephrologist per patient and sending it to an alternative information system, i.e. not transferred to the SNDS database, which we used in this study. Thus, we could expect to observe a decrease in the visit rate with nephrologists starting in 2020. However, preliminary analysis on the capitation payment model data shows that visits were still reported in the SNDS database. Conversely, the new model could incentivize a more regular follow-up and increase the observed visit rate. Assessing the effect of the new model is difficult due to the fact that a) no link is available between the data reported by the facilities and the SNDS database, which means that we cannot investigate the continuity of nephrology care at the individual level and b) the model was not adopted by all providers at the same time.

The main limitation of this study is the inability to determine the CKD stage of the study population, as the SNDS does not contain laboratory test results. This is important because healthcare utilization varies across CKD stages [34, 35] and the impact of COVID-19 likely varied as well [17]. The second limitation is selection bias inherent to the RENALGO algorithm, that relies on healthcare consumption data. Indeed, the sensitivity of the algorithm is poor for individuals who do not seek care or have not been diagnosed or whose care is inappropriate, which could ultimately underestimate the true extent of the pandemic-related disruptions.

Our study has several strengths. The data used come from an exhaustive source of in- and out-patient healthcare use for nearly the whole French population. The SNDS database also contains information on deaths, ascertained by certificate. This allowed calculating the mortality rates in the 2017 and 2019 cohorts in order to isolate the direct effect of COVID-19 as a disruptive event for the healthcare system.

By comparing the 2017 and 2019 cohorts, it was assumed that the COVID-19 pandemic was the only major contextual difference and the reason for the observed differences. However, this design cannot exclude the effect of other changes. For example, in 2020, France introduced a new capitation payment model to improve the care of individuals with stage 4 and 5 CKD. This could have affected how consultations with a nephrologist are provided and captured.

Our study focused on the quantitative aspect of healthcare utilization. The quality of care should also be investigated. This includes the effectiveness of teleconsultations in the context of CKD for which robust data are still lacking [36, 37].

## Conclusions

This study shows a reassuring continuity of outpatient care for individuals with CKD in France during the 2 years following the COVID-19 pandemic, notably thanks to teleconsultations. The persistent decrease in inpatient healthcare use warrants additional investigations.

## Supplementary Information

Below is the link to the electronic supplementary material.

Supplementary file1 (DOCX 6425 KB)

## Data Availability

The dataset analyzed in this study is not publicly available due to French law which forbids sharing data from a SNDS data extraction.
