# Supplementary material for: The evolution of consultation practices with general practitioners and nephrologists for patients with chronic kidney disease before and after the COVID-19 pandemic in France
Source: J Nephrol. 2025 Jul 27;38(8):2407–16. doi: 10.1007/s40620-025-02376-5 (PMC12630256; doi:10.1007/s40620-025-02376-5)

**Supplementary material**

**Table S1:** Sociodemographic characteristics and comorbidities of patients with CKD in France in 2017 and 2019

**Table S2:** Percentage of patients in each cohort who saw a GP at least once, a nephrologist at least once, and had at least one hospital stay, stratified by sex and age group in each period, in person-time, adjusted for deaths

**Table S3:** Percentage of patients in each cohort who saw a GP at least once, a nephrologist at least once and had at least one hospital stay, stratified on city-level deprivation index in each period, in person-time, adjusted for deaths

**Figure S1:** The COVID-19 pandemic periods in France

**Figure S2**: Flowchart for the 2017 (A) and 2019 (B) cohorts

**Figure S3:** Weekly death rates in the 2017 and 2019 cohorts during the two years of follow-up.

**Figure S4:** Percentages of patients with ≥ 1 GP consultation (A) and of patients with ≥ 1 nephrologist consultation (B) by cohort and by sex.

**Figure S5:** Percentages of patients with ≥ 1 GP consultation (A) and of patients with ≥ 1 nephrologist consultation (B) by cohort and by age group

**Figure S6:** Cumulative incidence of a first GP consultation in the 2017 and 2019 cohorts during the follow-up, by region

**Figure S7:** Cumulative incidence of a first nephrologist consultation in the 2017 and 2019 cohorts during the follow-up, by region

**Table S1:** Sociodemographic characteristics and comorbidities of patients with CKD in France in 2017 and 2019

|  | **2017 Cohort** | **2019 Cohort** |
| --- | --- | --- |
|  | **(N=4 866 096)** | **(N=5 089 706)** |
|  |  |  |
| **Age (median [Q1 – Q3])** | 67 [48 - 78] | 67 [49 – 78] |
| **Number of patients in the age group** |  |  |
| < 60 years | 1 813 280 (37.3%) | 1 840 746 (36.2%) |
| 60 – 74 years | 1 468 357 (30.2%) | 1 577 660 (31.0%) |
| ≥ 75 years | 1 584 459 (32.6%) | 1 671 300 (32.8%) |
| **Sex** |  |  |
| Men | 1 965 506 (40.4%) | 2 094 915 (41.2%) |
| Women | 2 900 590 (59.6%) | 2 994 791 (58.8%) |
| **Region of residence*** |  |  |
| Auvergne-Rhône-Alpes | 587 600 (12.1%) | 619 508 (12.2%) |
| Bourgogne-Franche-Comté | 210 014 (4.3%) | 224 823 (4.4%) |
| Bretagne | 209 501 (4.3%) | 227 316 (4.5%) |
| Centre-Val de Loire | 190 884 (3.9%) | 209 995 (4.1%) |
| Corse | 31 682 (0.7%) | 31 769 (0.6%) |
| Grand-Est | 463 114 (9.5%) | 491 778 (9.7%) |
| Hauts-de-France | 468 758 (9.6%) | 471 116 (9.3%) |
| Ile-de-France | 759 075 (15.6%) | 783 547 (15.4%) |
| Normandie | 241 100 (5.0%) | 265 462 (5.2%) |
| Nouvelle-Aquitaine | 436 121 (9.0%) | 447 879 (8.8%) |
| Occitanie | 470 754 (9.7%) | 477 608 (9.4%) |
| Overseas territories | 111 010 (2.3%) | 120 579 (2.4%) |
| Pays de la Loire | 233 952 (4.8%) | 254 559 (5.0%) |
| Provence-Alpes-Côte d’Azur | 451 054 (9.3%) | 461 786 (9.1%) |
| **City-level deprivation index (quintile)*** |  |  |
| 1 (more advantaged) | 853 832 (17.6%) | 891 545 (17.5%) |
| 2 | 901 212 (18.5%) | 941 052 (18.5%) |
| 3 | 970 267 (19.9%) | 1 011 197 (19.9%) |
| 4 | 974 859 (20.0%) | 1 017 363 (20.0%) |
| 5 (more disadvantaged) | 1 011 880 (20.8%) | 1 036 269 (20.3%) |
| **History of:** |  |  |
| Cardio-neurovascular diseases | 1 469 336 (30.2%) | 1 563 654 (30.7%) |
| Vascular risk management | 1 137 358 (23.4%) | 1 179 402 (23.2%) |
| Diabetes | 1 233 817 (25.4%) | 1 294 512 (25.4%) |
| Cancer | 9 799 66 (20.1%) | 1 067 996 (21.0%) |
| Psychiatric diseases | 384 030 (7.9%) | 424 819 (8.3%) |
| Psychotropic treatment without psychiatry diagnosis found | 1 011 969 (20.8%) | 996 182 (19.6%) |
| Neurological or degenerative diseases | 338 406 (7.0%) | 347 588 (6.8%) |
| Chronic respiratory diseases (excluding cystic fibrosis) | 611 847 (12.6%) | 644 915 (12.7%) |
| Inflammatory or rare diseases or HIV or AIDS | 432 464 (8.9%) | 461 545 (9.1%) |

* less than 4% missing

**Table S2:** Percentage of patients in each cohort who saw a GP at least once, a nephrologist at least once, and had at least one hospital stay, stratified by sex and age group in each period, in person-time, adjusted for deaths

|  |  |  | **% of patients with ≥ 1 GP consultation** | | | **% of patients with ≥ 1 nephrologist consultation** | | | | **% of patients with ≥ 1 hospital stay <24 h** | | | **% of patients with ≥ 1 hospital stay >24 h** | | |
| --- | --- | --- | --- | --- | --- | --- | --- | --- | --- | --- | --- | --- | --- | --- | --- |
|  |  |  | **2017**  **cohort** | **2019**  **cohort** | **Relative**  **difference** | **2017**  **cohort** | **2019**  **cohort** | **Relative**  **difference** | **2017**  **cohort** | | **2019**  **cohort** | **Relative**  **difference** | **2017**  **cohort** | **2019**  **cohort** | **Relative**  **difference** |
| **< 60 years** | **Women** | **Year N+1** | 88.9 | 87.0 | -2.1 | 2.9 | 2.9 | 0.0 | 20.6 | | 19.8 | -3.9 | 24.2 | 23.0 | -5.0 |
|  |  | **Year N+2** | 86.4 | 86.4 | 0.0 | 2.6 | 2.8 | 7.7 | 18.6 | | 19.9 | 7.0 | 12.5 | 12.3 | -1.6 |
|  |  | **Pre-pandemic** | 59.5 | 58.8 | -1.2 | 1.1 | 1.1 | 0.0 | 8.2 | | 8.7 | 6.1 | 11.3 | 11.2 | -0.9 |
|  |  | **1^st^ lockdown** | 59.6 | 61.5 | 3.2 | 1.1 | 1.3 | 18.2 | 5.8 | | 4.0 | -31.0 | 6.2 | 5.4 | -12.9 |
|  |  | **Post-1^st^ lockdown** | 74.9 | 72.9 | -2.7 | 1.8 | 1.9 | 5.6 | 11.3 | | 11.6 | 2.7 | 7.2 | 6.6 | -8.3 |
|  |  | **2^nd^ lockdown** | 43.8 | 40.7 | -7.1 | 0.7 | 0.6 | -14.3 | 4.7 | | 4.7 | 0.0 | 1.9 | 1.6 | -15.8 |
|  |  | **Post-2^nd^ lockdown** | 66.1 | 63.1 | -4.5 | 1.2 | 1.3 | 8.3 | 8.0 | | 8.8 | 10.0 | 4.2 | 3.9 | -7.1 |
|  |  | **3^rd^ lockdown** | 29.4 | 29.0 | -1.4 | 0.4 | 0.4 | 0.0 | 3.2 | | 3.3 | 3.1 | 1.3 | 1.2 | -7.7 |
|  |  | **Post-3^rd^ lockdown** | 35.1 | 34.7 | -1.1 | 0.5 | 0.6 | 20.0 | 3.8 | | 4.2 | 10.5 | 1.6 | 1.6 | 0.0 |
|  |  | **Post-pandemic** | 75.5 | 77.0 | 2.0 | 1.8 | 2.0 | 11.1 | 11.8 | | 12.8 | 8.5 | 7.6 | 7.6 | 0.0 |
|  | **Men** | **Year N+1** | 90.3 | 89.4 | -1.0 | 7.3 | 7.4 | 1.4 | 24.6 | | 22.7 | -7.7 | 16.9 | 15.0 | -11.2 |
|  |  | **Year N+2** | 88.2 | 87.9 | -0.3 | 7.0 | 7.6 | 8.6 | 22.8 | | 23.5 | 3.1 | 13.9 | 12.7 | -8.6 |
|  |  | **Pre-pandemic** | 64.5 | 63.3 | -1.9 | 2.9 | 2.9 | 0.0 | 8.1 | | 8.5 | 4.9 | 6.3 | 5.9 | -6.3 |
|  |  | **1^st^ lockdown** | 64.8 | 65.9 | 1.7 | 2.9 | 3.4 | 17.2 | 5.7 | | 3.1 | -45.6 | 4.0 | 2.6 | -35.0 |
|  |  | **Post-1^st^ lockdown** | 78.6 | 78.3 | -0.4 | 4.8 | 4.9 | 2.1 | 13.1 | | 13.0 | -0.8 | 9.0 | 8.2 | -8.9 |
|  |  | **2^nd^ lockdown** | 47.3 | 45.0 | -4.9 | 1.9 | 1.8 | -5.3 | 5.0 | | 4.6 | -8.0 | 3.0 | 2.5 | -16.7 |
|  |  | **Post-2^nd^ lockdown** | 70.1 | 68.1 | -2.9 | 3.4 | 3.6 | 5.9 | 9.1 | | 9.2 | 1.1 | 5.9 | 5.2 | -11.9 |
|  |  | **3^rd^ lockdown** | 31.7 | 32.0 | 0.9 | 1.0 | 1.1 | 10.0 | 3.2 | | 3.3 | 3.1 | 1.8 | 1.6 | -11.1 |
|  |  | **Post-3^rd^ lockdown** | 38.2 | 38.3 | 0.3 | 1.4 | 1.5 | 7.1 | 3.9 | | 4.3 | 10.3 | 2.3 | 2.1 | -8.7 |
|  |  | **Post-pandemic** | 79.7 | 79.7 | 0.0 | 5.1 | 5.6 | 9.8 | 13.8 | | 14.9 | 8.0 | 8.5 | 7.7 | -9.4 |
| **61-74 years** | **Women** | **Year N+1** | 97.5 | 95.1 | -2.5 | 6.2 | 6.0 | -3.2 | 31.0 | | 28.6 | -7.7 | 21.5 | 18.6 | -13.5 |
|  |  | **Year N+2** | 94.5 | 93.3 | -1.3 | 6.0 | 6.2 | 3.3 | 28.0 | | 28.7 | 2.5 | 18.3 | 16.6 | -9.3 |
|  |  | **Pre-pandemic** | 79.9 | 76.8 | -3.9 | 2.2 | 2.3 | 4.5 | 12.5 | | 13.0 | 4.0 | 7.4 | 6.9 | -6.8 |
|  |  | **1^st^ lockdown** | 80.4 | 78.9 | -1.9 | 2.3 | 2.6 | 13.0 | 9.0 | | 5.5 | -38.9 | 4.8 | 2.9 | -39.6 |
|  |  | **Post-1^st^ lockdown** | 92.2 | 89.9 | -2.5 | 4.0 | 4.0 | 0.0 | 17.4 | | 17.5 | 0.6 | 11.2 | 10.1 | -9.8 |
|  |  | **2^nd^ lockdown** | 62.9 | 57.5 | -8.6 | 1.6 | 1.4 | -12.5 | 7.6 | | 7.3 | -3.9 | 3.9 | 3.1 | -20.5 |
|  |  | **Post-2^nd^ lockdown** | 85.0 | 83.5 | -1.8 | 2.8 | 2.9 | 3.6 | 12.5 | | 13.0 | 4.0 | 7.7 | 6.5 | -15.6 |
|  |  | **3^rd^ lockdown** | 42.1 | 41.0 | -2.6 | 0.9 | 0.8 | -11.1 | 5.1 | | 5.2 | 2.0 | 2.3 | 2.0 | -13.0 |
|  |  | **Post-3^rd^ lockdown** | 51.2 | 50.6 | -1.2 | 1.2 | 1.2 | 0.0 | 6.0 | | 6.5 | 8.3 | 2.9 | 2.6 | -10.3 |
|  |  | **Post-pandemic** | 88.7 | 89.4 | 0.8 | 4.3 | 4.5 | 4.7 | 17.5 | | 18.7 | 6.9 | 10.9 | 10.1 | -7.3 |
|  | **Men** | **Year N+1** | 97.4 | 96.9 | -0.5 | 9.3 | 9.1 | -2.2 | 33.9 | | 32.6 | -3.8 | 27.1 | 24.9 | -8.1 |
|  |  | **Year N+2** | 96.2 | 96.4 | 0.2 | 9.5 | 9.7 | 2.1 | 31.8 | | 33.0 | 3.8 | 24.0 | 22.3 | -7.1 |
|  |  | **Pre-pandemic** | 77.1 | 75.6 | -1.9 | 3.5 | 3.5 | 0.0 | 13.2 | | 14.2 | 7.6 | 9.8 | 9.4 | -4.1 |
|  |  | **1^st^ lockdown** | 77.9 | 78.0 | 0.1 | 3.5 | 4.0 | 14.3 | 9.6 | | 6.1 | -36.5 | 6.3 | 4.3 | -31.7 |
|  |  | **Post-1^st^ lockdown** | 91.0 | 90.9 | -0.1 | 6.3 | 6.2 | -1.6 | 19.1 | | 19.9 | 4.2 | 14.5 | 13.7 | -5.5 |
|  |  | **2^nd^ lockdown** | 59.4 | 55.9 | -5.9 | 2.6 | 2.3 | -11.5 | 8.3 | | 8.3 | 0.0 | 5.3 | 4.4 | -17.0 |
|  |  | **Post-2^nd^ lockdown** | 84.6 | 85.0 | 0.5 | 4.5 | 4.5 | 0.0 | 14.0 | | 15.0 | 7.1 | 10.3 | 9.2 | -10.7 |
|  |  | **3^rd^ lockdown** | 39.6 | 38.4 | -3.0 | 1.4 | 1.3 | -7.1 | 5.7 | | 6.0 | 5.3 | 3.3 | 2.9 | -12.1 |
|  |  | **Post-3^rd^ lockdown** | 48.6 | 48.8 | 0.4 | 2.0 | 2.0 | 0.0 | 6.7 | | 7.4 | 10.4 | 4.0 | 3.7 | -7.5 |
|  |  | **Post-pandemic** | 91.9 | 91.8 | -0.1 | 7.2 | 7.2 | 0.0 | 20.4 | | 21.7 | 6.4 | 14.8 | 13.7 | -7.4 |
| **≥ 75 years** | **Women** | **Year N+1** | 99.5 | 97.9 | -1.6 | 5.6 | 5.5 | -1.8 | 24.6 | | 22.7 | -7.7 | 32.8 | 29.0 | -11.6 |
|  |  | **Year N+2** | 95.6 | 95.1 | -0.5 | 5.4 | 5.5 | 1.9 | 22.8 | | 23.5 | 3.1 | 29.7 | 26.5 | -10.8 |
|  |  | **Pre-pandemic** | 85.4 | 82.2 | -3.7 | 2.0 | 2.1 | 5.0 | 8.1 | | 8.5 | 4.9 | 10.7 | 9.9 | -7.5 |
|  |  | **1^st^ lockdown** | 87.2 | 85.3 | -2.2 | 2.0 | 2.4 | 20.0 | 5.7 | | 3.1 | -45.6 | 7.1 | 4.8 | -32.4 |
|  |  | **Post-1^st^ lockdown** | 93.9 | 92.3 | -1.7 | 3.7 | 3.7 | 0.0 | 13.1 | | 13.0 | -0.8 | 17.4 | 15.7 | -9.8 |
|  |  | **2^nd^ lockdown** | 68.4 | 63.4 | -7.3 | 1.4 | 1.2 | -14.3 | 5.0 | | 4.6 | -8.0 | 5.9 | 4.7 | -20.3 |
|  |  | **Post-2^nd^ lockdown** | 88.0 | 86.9 | -1.2 | 2.5 | 2.5 | 0.0 | 9.1 | | 9.2 | 1.1 | 12.5 | 10.3 | -17.6 |
|  |  | **3^rd^ lockdown** | 49.3 | 46.6 | -5.5 | 0.7 | 0.7 | 0.0 | 3.2 | | 3.3 | 3.1 | 3.7 | 3.1 | -16.2 |
|  |  | **Post-3^rd^ lockdown** | 58.4 | 56.5 | -3.3 | 1.0 | 1.0 | 0.0 | 3.9 | | 4.3 | 10.3 | 4.5 | 4.0 | -11.1 |
|  |  | **Post-pandemic** | 89.8 | 89.8 | 0.0 | 3.9 | 4.0 | 2.6 | 13.8 | | 14.9 | 8.0 | 17.9 | 16.3 | -8.9 |
|  | **Men** | **Year N+1** | 101.0 | 101.0 | 0.0 | 10.3 | 10.2 | -1.0 | 32.1 | | 31.0 | -3.4 | 38.8 | 35.8 | -7.7 |
|  |  | **Year N+2** | 98.6 | 99.4 | 0.8 | 10.5 | 10.7 | 1.9 | 30.6 | | 32.0 | 4.6 | 35.6 | 32.8 | -7.9 |
|  |  | **Pre-pandemic** | 83.8 | 82.1 | -2.0 | 3.9 | 3.9 | 0.0 | 11.2 | | 12.0 | 7.1 | 13.4 | 12.8 | -4.5 |
|  |  | **1^st^ lockdown** | 86.1 | 85.8 | -0.3 | 4.0 | 4.6 | 15.0 | 7.9 | | 4.8 | -39.2 | 8.9 | 6.5 | -27.0 |
|  |  | **Post-1^st^ lockdown** | 94.2 | 94.4 | 0.2 | 7.1 | 7.1 | 0.0 | 17.5 | | 18.3 | 4.6 | 20.8 | 19.7 | -5.3 |
|  |  | **2^nd^ lockdown** | 66.4 | 62.8 | -5.4 | 2.8 | 2.5 | -10.7 | 7.1 | | 7.0 | -1.4 | 7.5 | 6.4 | -14.7 |
|  |  | **Post-2^nd^ lockdown** | 88.8 | 89.1 | 0.3 | 5.0 | 5.1 | 2.0 | 12.7 | | 13.3 | 4.7 | 15.4 | 13.4 | -13.0 |
|  |  | **3^rd^ lockdown** | 47.1 | 44.7 | -5.1 | 1.6 | 1.4 | -12.5 | 4.8 | | 5.1 | 6.2 | 4.8 | 4.1 | -14.6 |
|  |  | **Post-3^rd^ lockdown** | 56.4 | 55.4 | -1.8 | 2.2 | 2.2 | 0.0 | 5.7 | | 6.4 | 12.3 | 5.9 | 5.4 | -8.5 |
|  |  | **Post-pandemic** | 93.5 | 93.7 | 0.2 | 7.9 | 8.1 | 2.5 | 19.2 | | 20.8 | 8.3 | 22.0 | 20.3 | -7.7 |

**Table S3:** Percentage of patients in each cohort who saw a GP at least once, a nephrologist at least once and had at least one hospital stay, stratified on city-level deprivation index in each period, in person-time, adjusted for deaths

|  |  | **% of patients with ≥ 1 GP consultation** | | | **% of patients with ≥ 1 nephrologist consultation** | | | | **% of patients with ≥ 1 hospital stay < 24 h** | | | **% of patients with ≥ 1 hospital stay >24 h** | | |
| --- | --- | --- | --- | --- | --- | --- | --- | --- | --- | --- | --- | --- | --- | --- |
|  |  | **2017**  **cohort** | **2019**  **cohort** | **Relative**  **difference** | **2017**  **cohort** | **2019**  **cohort** | **Relative**  **difference** | **2017**  **cohort** | | **2019**  **cohort** | **Relative**  **difference** | **2017**  **cohort** | **2019**  **cohort** | **Relative**  **difference** |
| **1** | **Year N+1** | 92.7 | 91.5 | -1.2 | 5.7 | 5.7 | 0.0 | 28.2 | | 26.3 | -1.9 | 25.8 | 23.6 | -2.2 |
|  | **Year N+2** | 90.0 | 90.1 | 0.1 | 5.5 | 6.3 | 0.8 | 25.7 | | 26.6 | 0.9 | 19.7 | 18.3 | -1.4 |
|  | **Period 1** | 67.4 | 66.2 | -1.2 | 2.1 | 2.2 | 0.1 | 10.9 | | 11.3 | 0.4 | 9.8 | 9.3 | -0.5 |
|  | **Period 2** | 68.1 | 69.4 | 1.3 | 2.1 | 2.5 | 0.4 | 7.8 | | 4.7 | -3.1 | 6.1 | 4.5 | -1.6 |
|  | **Period 3** | 82.0 | 81.1 | -0.9 | 3.7 | 3.8 | 0.1 | 15.6 | | 15.7 | 0.1 | 11.9 | 11.1 | -0.8 |
|  | **Period 4** | 51.7 | 47.6 | -4.1 | 1.5 | 1.4 | -0.1 | 6.6 | | 6.4 | -0.2 | 4.0 | 3.3 | -0.7 |
|  | **Period 5** | 73.5 | 72.5 | -1.0 | 2.6 | 2.8 | 0.2 | 11.2 | | 11.8 | 0.6 | 8.1 | 7.1 | -1.0 |
|  | **Period 6** | 34.4 | 33.4 | -1.0 | 0.8 | 0.8 | 0.0 | 4.4 | | 4.5 | 0.1 | 2.5 | 2.1 | -0.4 |
|  | **Period 7** | 41.7 | 41.3 | -0.4 | 1.1 | 1.2 | 0.1 | 5.3 | | 5.7 | 0.4 | 3.1 | 2.8 | -0.3 |
|  | **Period 8** | 81.5 | 82.2 | 0.7 | 4.0 | 4.7 | 0.7 | 16.1 | | 17.3 | 1.2 | 12.0 | 11.2 | -0.8 |
| **2** | **Year N+1** | 95.2 | 94.1 | -1.1 | 6.4 | 6.4 | 0.0 | 27.4 | | 26.3 | -1.1 | 26.3 | 24.2 | -2.1 |
|  | **Year N+2** | 92.6 | 92.7 | 0.1 | 6.2 | 6.5 | 0.3 | 25.2 | | 26.5 | 1.3 | 20.2 | 18.9 | -1.3 |
|  | **Period 1** | 73.2 | 71.7 | -1.5 | 2.4 | 2.5 | 0.1 | 10.5 | | 11.2 | 0.7 | 9.9 | 9.5 | -0.4 |
|  | **Period 2** | 74.0 | 74.6 | 0.6 | 2.4 | 2.8 | 0.4 | 7.5 | | 4.8 | -2.7 | 6.1 | 4.5 | -1.6 |
|  | **Period 3** | 86.1 | 85.2 | -0.9 | 4.2 | 4.3 | 0.1 | 15.3 | | 15.7 | 0.4 | 12.2 | 11.4 | -0.8 |
|  | **Period 4** | 56.2 | 52.4 | -3.8 | 1.7 | 1.5 | -0.2 | 6.4 | | 6.3 | -0.1 | 4.1 | 3.4 | -0.7 |
|  | **Period 5** | 78.5 | 77.5 | -1.0 | 2.9 | 3.0 | 0.1 | 10.9 | | 11.7 | 0.8 | 8.4 | 7.3 | -1.1 |
|  | **Period 6** | 38.2 | 37.1 | -1.1 | 0.9 | 0.8 | -0.1 | 4.3 | | 4.5 | 0.2 | 2.5 | 2.2 | -0.3 |
|  | **Period 7** | 45.9 | 45.5 | -0.4 | 1.2 | 1.3 | 0.1 | 5.1 | | 5.7 | 0.6 | 3.1 | 2.9 | -0.2 |
|  | **Period 8** | 85.3 | 86.0 | 0.7 | 4.5 | 4.8 | 0.3 | 15.9 | | 17.2 | 1.3 | 12.3 | 11.6 | -0.7 |
| **3** | **Year N+1** | 95.7 | 94.5 | -1.2 | 6.4 | 6.3 | -0.1 | 27.2 | | 26.2 | -1.0 | 27.2 | 24.7 | -2.5 |
|  | **Year N+2** | 93.1 | 93.0 | -0.1 | 6.2 | 6.4 | 0.2 | 25.1 | | 26.4 | 1.3 | 21.5 | 19.9 | -1.6 |
|  | **Period 1** | 74.9 | 73.3 | -1.6 | 2.5 | 2.5 | 0.0 | 10.4 | | 11.1 | 0.7 | 10.2 | 9.7 | -0.5 |
|  | **Period 2** | 75.7 | 76.0 | 0.3 | 2.5 | 2.8 | 0.3 | 7.4 | | 4.8 | -2.6 | 6.3 | 4.6 | -1.7 |
|  | **Period 3** | 87.1 | 86.1 | -1.0 | 4.3 | 4.3 | 0.0 | 15.1 | | 15.6 | 0.5 | 12.9 | 12.0 | -0.9 |
|  | **Period 4** | 57.6 | 54.0 | -3.6 | 1.7 | 1.5 | -0.2 | 6.3 | | 6.2 | -0.1 | 4.4 | 3.6 | -0.8 |
|  | **Period 5** | 80.0 | 78.8 | -1.2 | 3.0 | 3.0 | 0.0 | 10.9 | | 11.6 | 0.7 | 8.9 | 7.7 | -1.2 |
|  | **Period 6** | 39.5 | 38.4 | -1.1 | 0.9 | 0.9 | 0.0 | 4.3 | | 4.5 | 0.2 | 2.7 | 2.4 | -0.3 |
|  | **Period 7** | 47.6 | 47.0 | -0.6 | 1.3 | 1.3 | 0.0 | 5.1 | | 5.6 | 0.5 | 3.4 | 3.1 | -0.3 |
|  | **Period 8** | 86.1 | 86.7 | 0.6 | 4.6 | 4.7 | 0.1 | 15.9 | | 17.2 | 1.3 | 13.1 | 12.2 | -0.9 |
| **4** | **Year N+1** | 96.0 | 94.9 | -1.1 | 6.2 | 6.2 | 0.0 | 27.1 | | 26.0 | -1.1 | 27.7 | 25.3 | -2.4 |
|  | **Year N+2** | 93.2 | 93.2 | 0.0 | 6.0 | 6.2 | 0.2 | 24.9 | | 26.1 | 1.2 | 21.9 | 20.3 | -1.6 |
|  | **Period 1** | 75.9 | 74.1 | -1.8 | 2.3 | 2.3 | 0.0 | 10.3 | | 11.1 | 0.8 | 10.4 | 9.9 | -0.5 |
|  | **Period 2** | 76.8 | 76.7 | -0.1 | 2.3 | 2.7 | 0.4 | 7.4 | | 4.7 | -2.7 | 6.4 | 4.6 | -1.8 |
|  | **Period 3** | 87.7 | 86.8 | -0.9 | 4.1 | 4.2 | 0.1 | 15.0 | | 15.6 | 0.6 | 13.3 | 12.4 | -0.9 |
|  | **Period 4** | 57.9 | 54.5 | -3.4 | 1.6 | 1.4 | -0.2 | 6.3 | | 6.2 | -0.1 | 4.5 | 3.7 | -0.8 |
|  | **Period 5** | 80.8 | 79.6 | -1.2 | 2.8 | 2.9 | 0.1 | 10.7 | | 11.5 | 0.8 | 9.1 | 7.9 | -1.2 |
|  | **Period 6** | 39.6 | 38.5 | -1.1 | 0.9 | 0.8 | -0.1 | 4.2 | | 4.5 | 0.3 | 2.8 | 2.4 | -0.4 |
|  | **Period 7** | 47.8 | 47.3 | -0.5 | 1.2 | 1.2 | 0.0 | 5.0 | | 5.6 | 0.6 | 3.4 | 3.2 | -0.2 |
|  | **Period 8** | 86.3 | 87.0 | 0.7 | 4.4 | 4.5 | 0.1 | 15.7 | | 17.0 | 1.3 | 13.3 | 12.5 | -0.8 |
| **5** | **Year N+1** | 96.3 | 95.0 | -1.3 | 6.7 | 6.7 | 0.0 | 27.2 | | 25.8 | -1.4 | 28.2 | 25.7 | -2.5 |
|  | **Year N+2** | 93.2 | 93.2 | 0.0 | 6.5 | 6.7 | 0.2 | 24.9 | | 26.1 | 1.2 | 22.7 | 21.1 | -1.6 |
|  | **Period 1** | 77.6 | 75.6 | -2.0 | 2.5 | 2.6 | 0.1 | 10.3 | | 11.0 | 0.7 | 10.5 | 10.1 | -0.4 |
|  | **Period 2** | 78.5 | 77.9 | -0.6 | 2.5 | 3.0 | 0.5 | 7.3 | | 4.6 | -2.7 | 6.6 | 4.6 | -2.0 |
|  | **Period 3** | 88.6 | 87.4 | -1.2 | 4.5 | 4.6 | 0.1 | 15.1 | | 15.5 | 0.4 | 13.7 | 12.7 | -1.0 |
|  | **Period 4** | 60.2 | 56.4 | -3.8 | 1.8 | 1.5 | -0.3 | 6.3 | | 6.1 | -0.2 | 4.6 | 3.9 | -0.7 |
|  | **Period 5** | 81.9 | 80.6 | -1.3 | 3.1 | 3.2 | 0.1 | 10.8 | | 11.4 | 0.6 | 9.5 | 8.3 | -1.2 |
|  | **Period 6** | 41.8 | 40.5 | -1.3 | 1.0 | 0.9 | -0.1 | 4.2 | | 4.4 | 0.2 | 2.9 | 2.5 | -0.4 |
|  | **Period 7** | 49.8 | 49.2 | -0.6 | 1.3 | 1.3 | 0.0 | 5.0 | | 5.5 | 0.5 | 3.6 | 3.3 | -0.3 |
|  | **Period 8** | 86.2 | 87.3 | 1.1 | 4.8 | 4.9 | 0.1 | 15.8 | | 17.0 | 1.2 | 13.8 | 13.0 | -0.8 |

First lockdown

Second lockdown

Third lockdown

17/03

10/05

30/10

14/12

03/04

02/05

Post-pandemic

Pre-

pandemic

2020

2021

10/06

01/01

31/12

Period 1 Period 2 Period 3 Period 4 Period 5 Period 6 Period 7 Period 8

**Figure S1:** The COVID-19 pandemic periods in France

Patients with CKD untreated by renal replacement therapy in 2019 (identified by RENALGO algorithm)

N = 5 394 254

Patients with age and sex information

N = 5 304 924

Patients alive at 31/12/2019

N = 5 089 706

Patients with age and sex unknown

N = 89 330

Deceased patients before 01/01/2020

N = 215 218

Patients with CKD untreated by renal replacement therapy in 2017 (identified by RENALGO algorithm)

N = 5 186 477

Patients with age and sex information

N = 5 065 309

Patients alive at 31/12/2017

N = 4 866 096

Patients with age and sex unknown

N = 121 168

Deceased patients before 01/01/2018

N = 199 213

A. 2017 Cohort B. 2019 Cohort

**Figure S2**: Flowchart for the 2017 (A) and 2019 (B) cohorts.


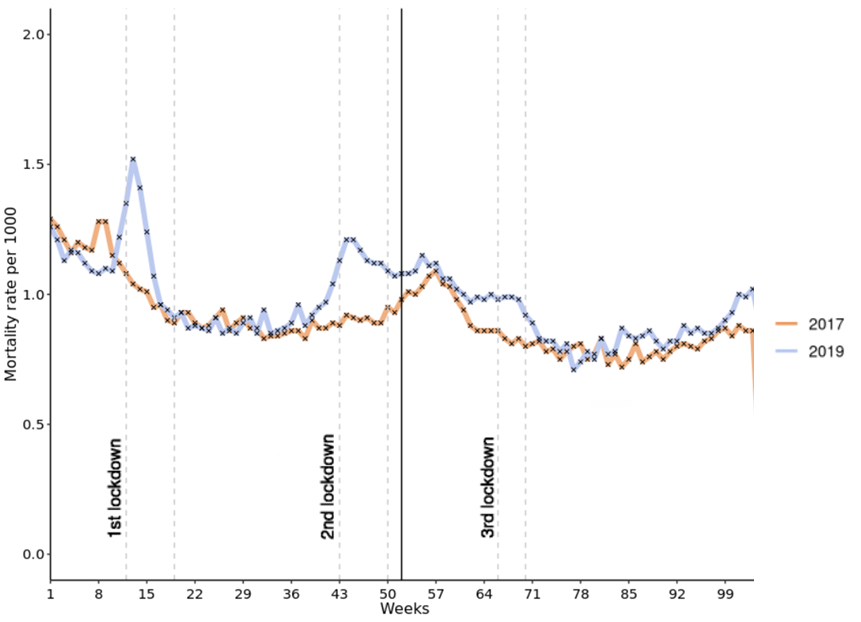


**Figure S3:** Weekly death rates in the 2017 and 2019 cohorts during the two years of follow-up.

**
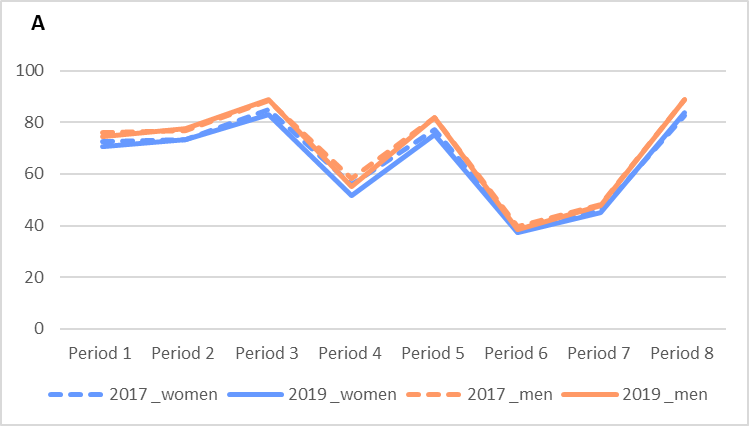
**

**
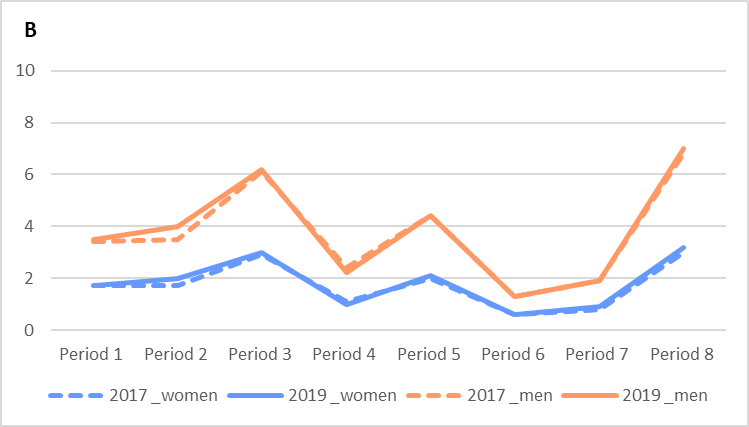
**

**Figure S4:** Percentages of patients with ≥ 1 GP consultation (A) and of patients with ≥ 1 nephrologist consultation (B) by cohort and by sex.

**
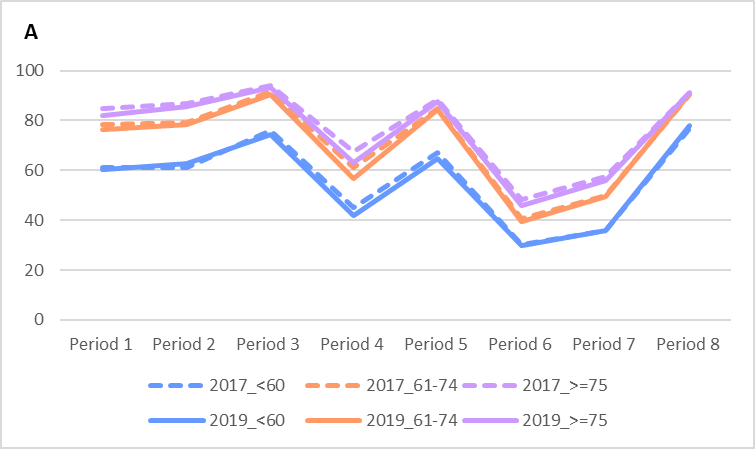
**

**
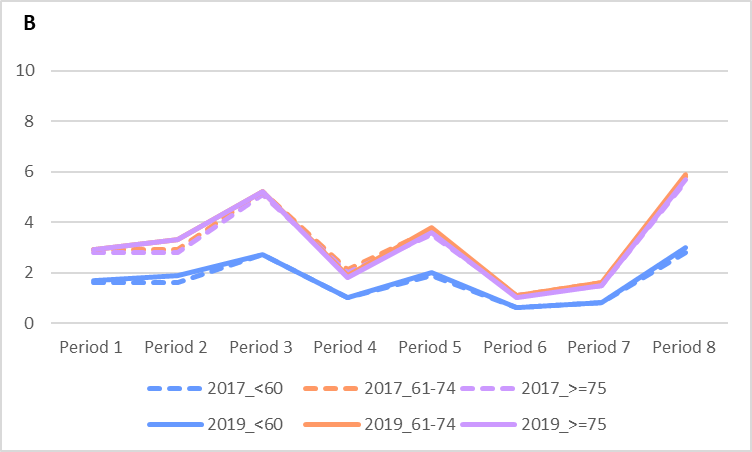
**

**Figure S5:** Percentages of patients with ≥ 1 GP consultation (A) and of patients with ≥ 1 nephrologist consultation (B) by cohort and by age group.

**Figure S6:** Cumulative incidence of a first GP consultation in the 2017 and 2019 cohorts during the follow-up, by region


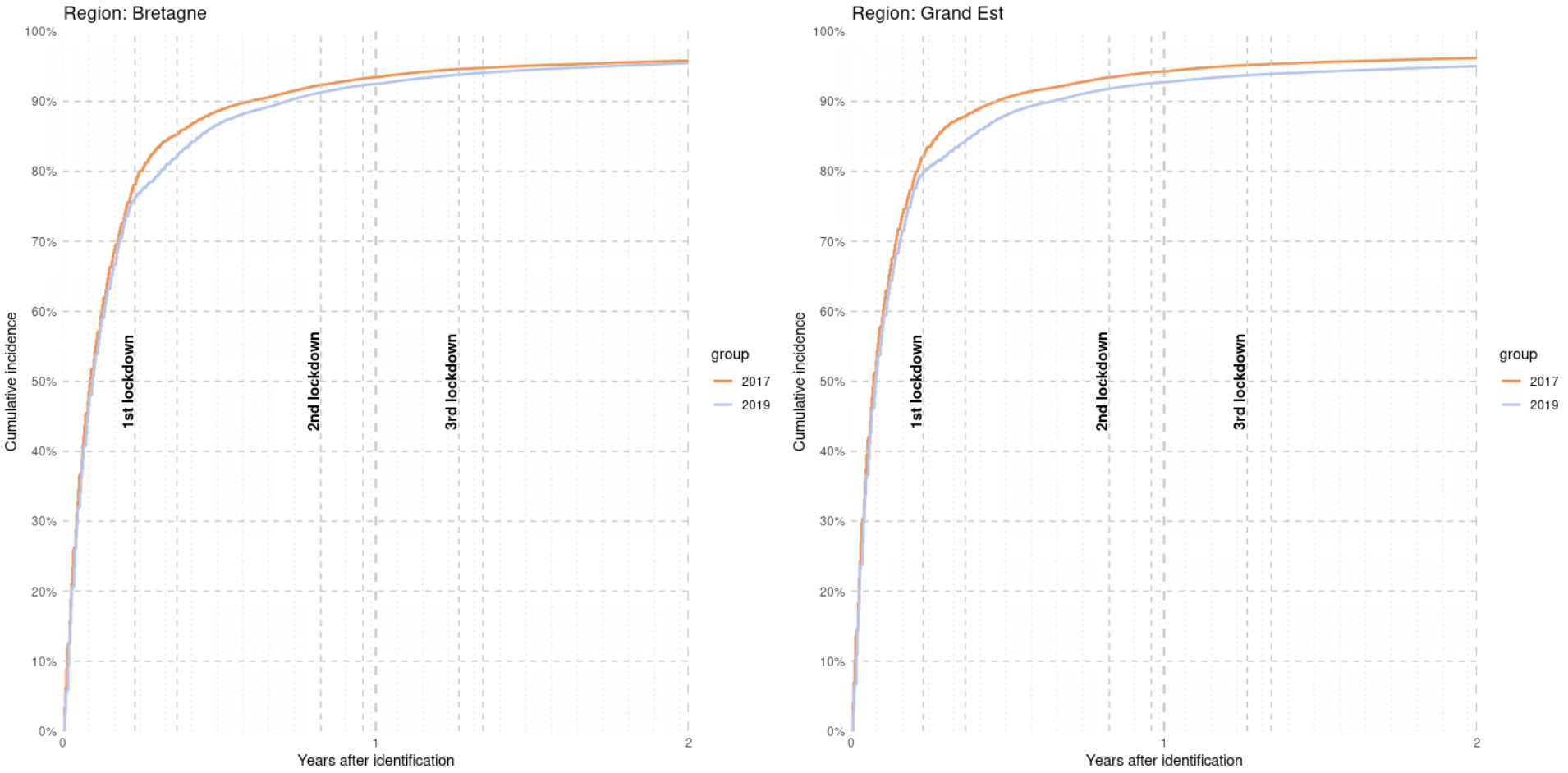


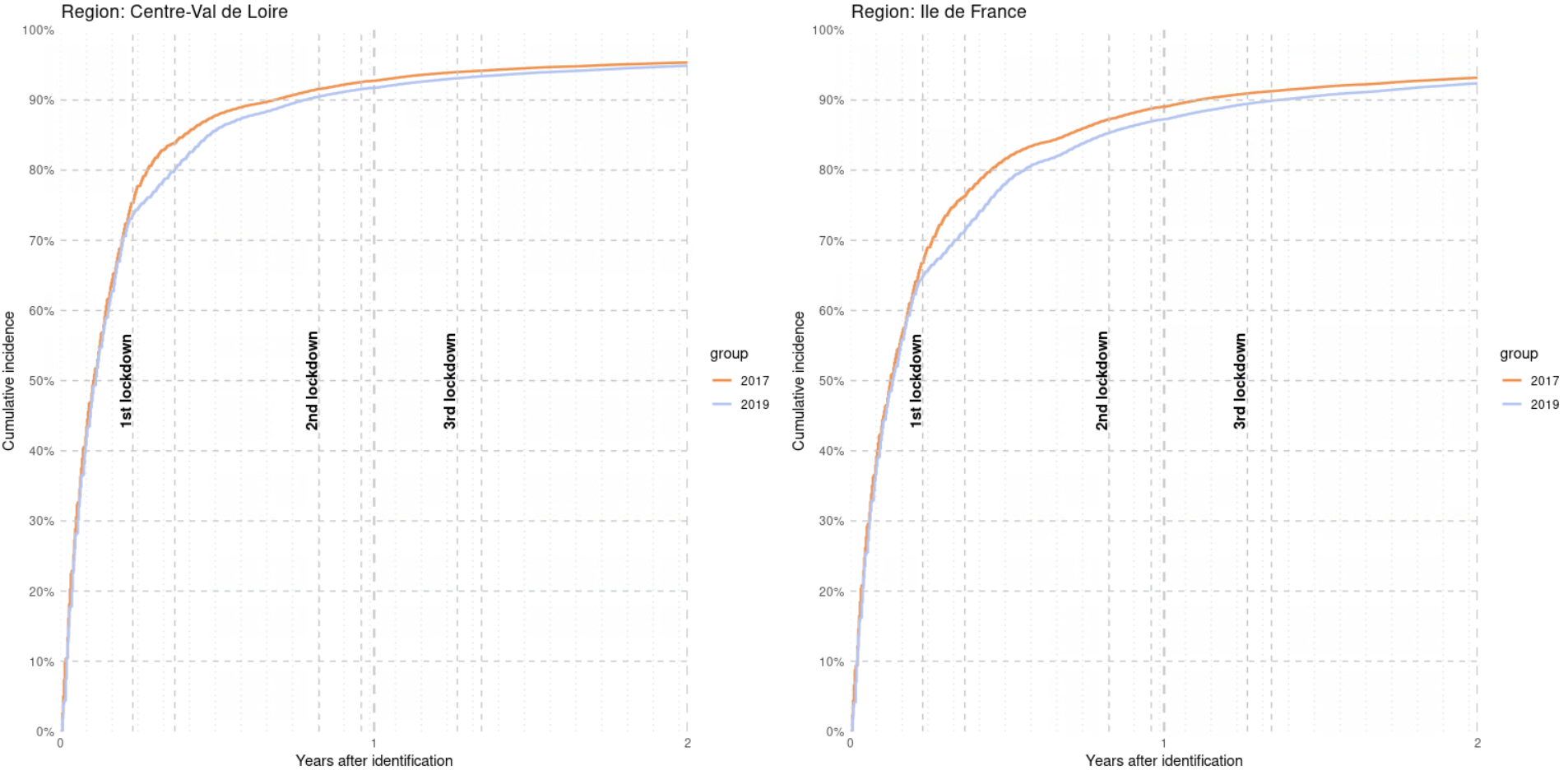


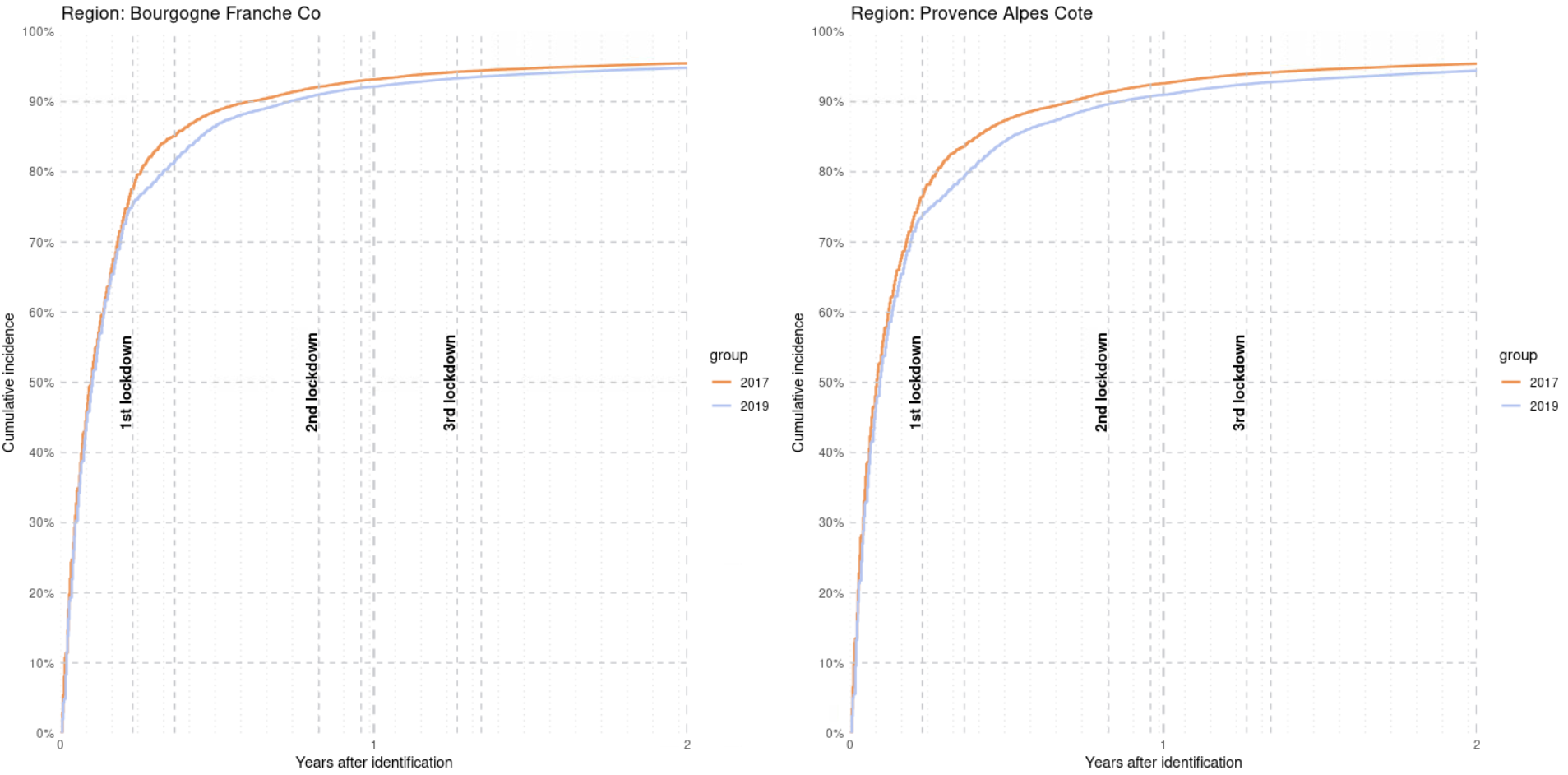


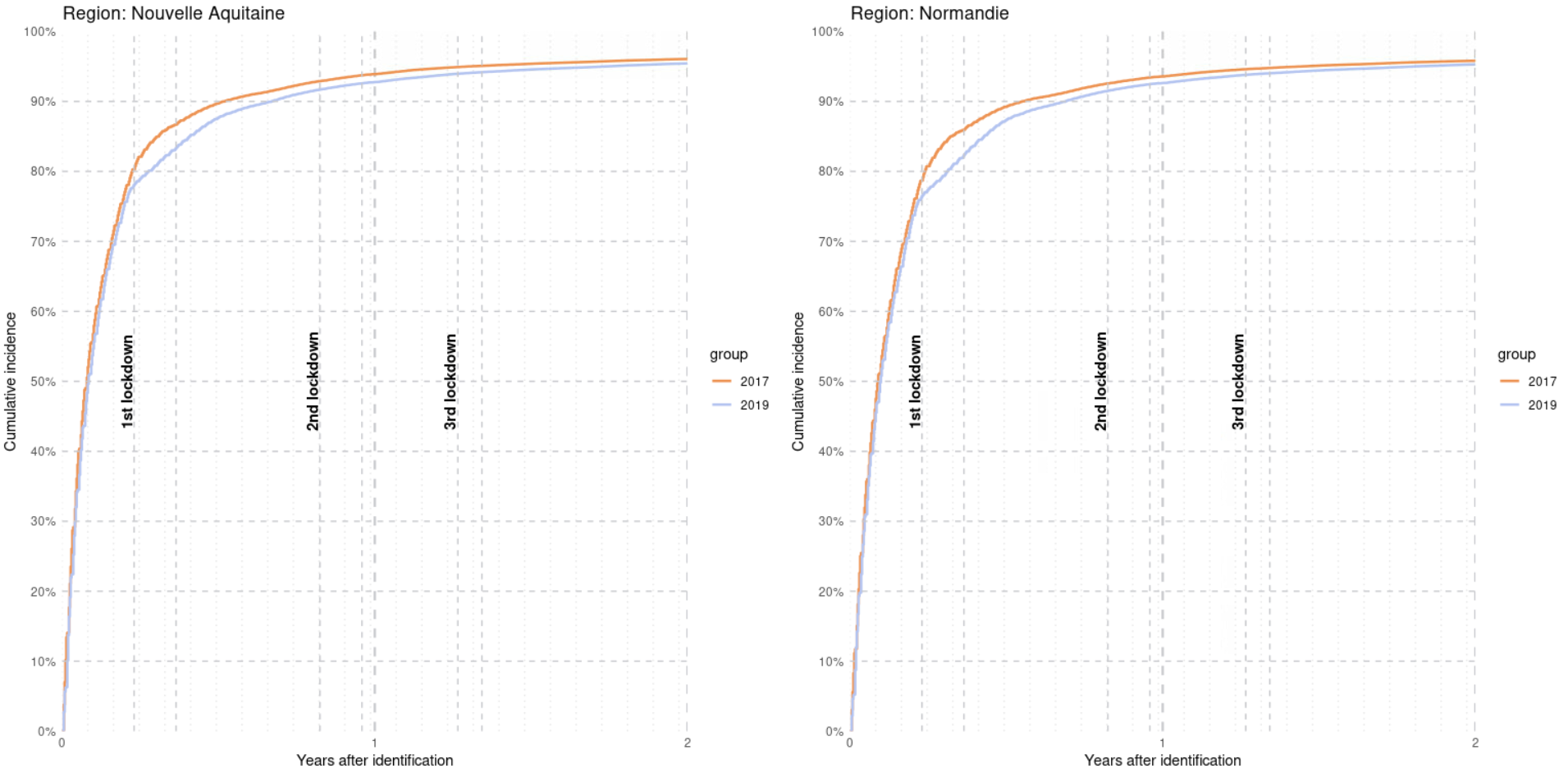


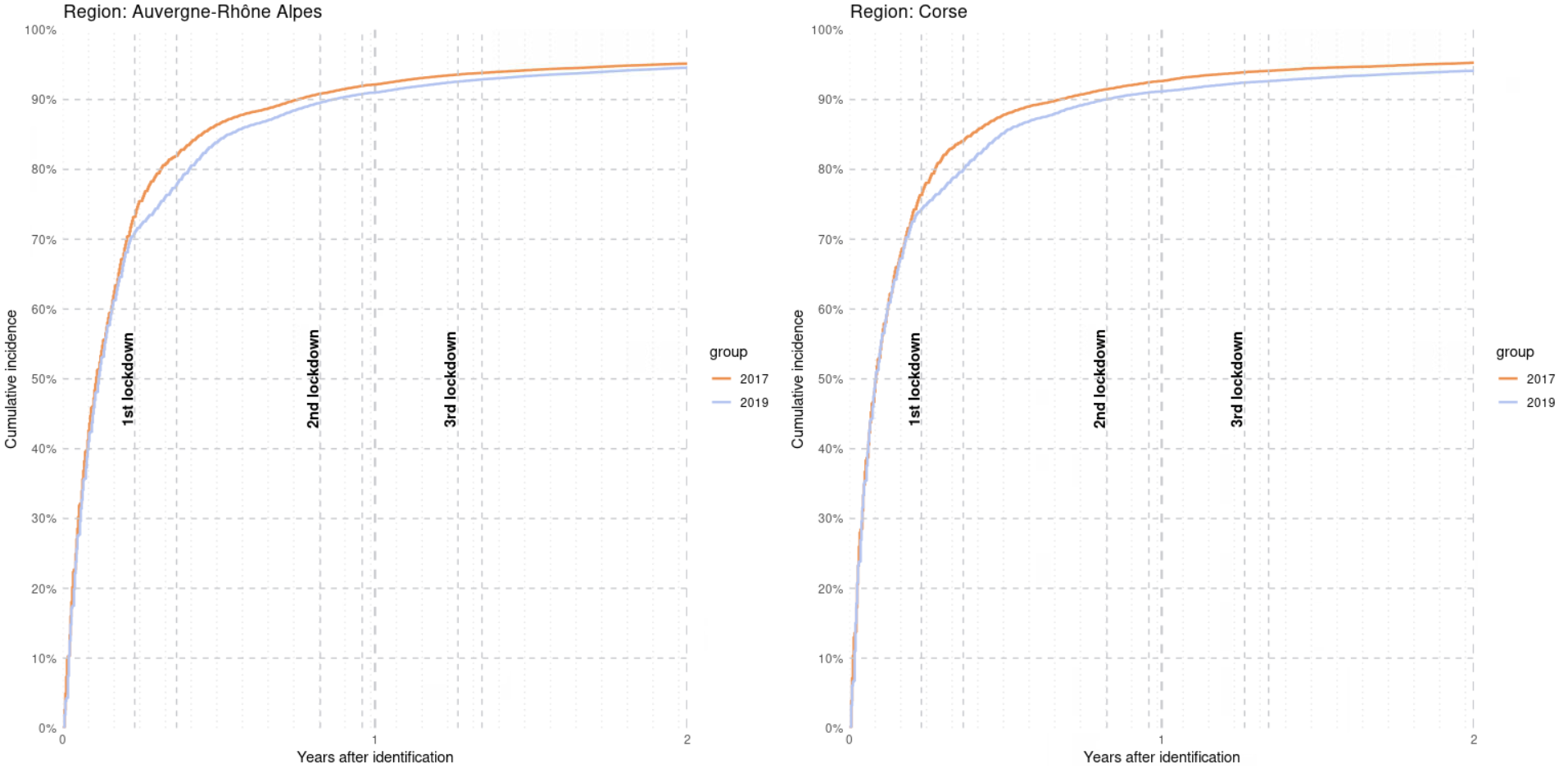


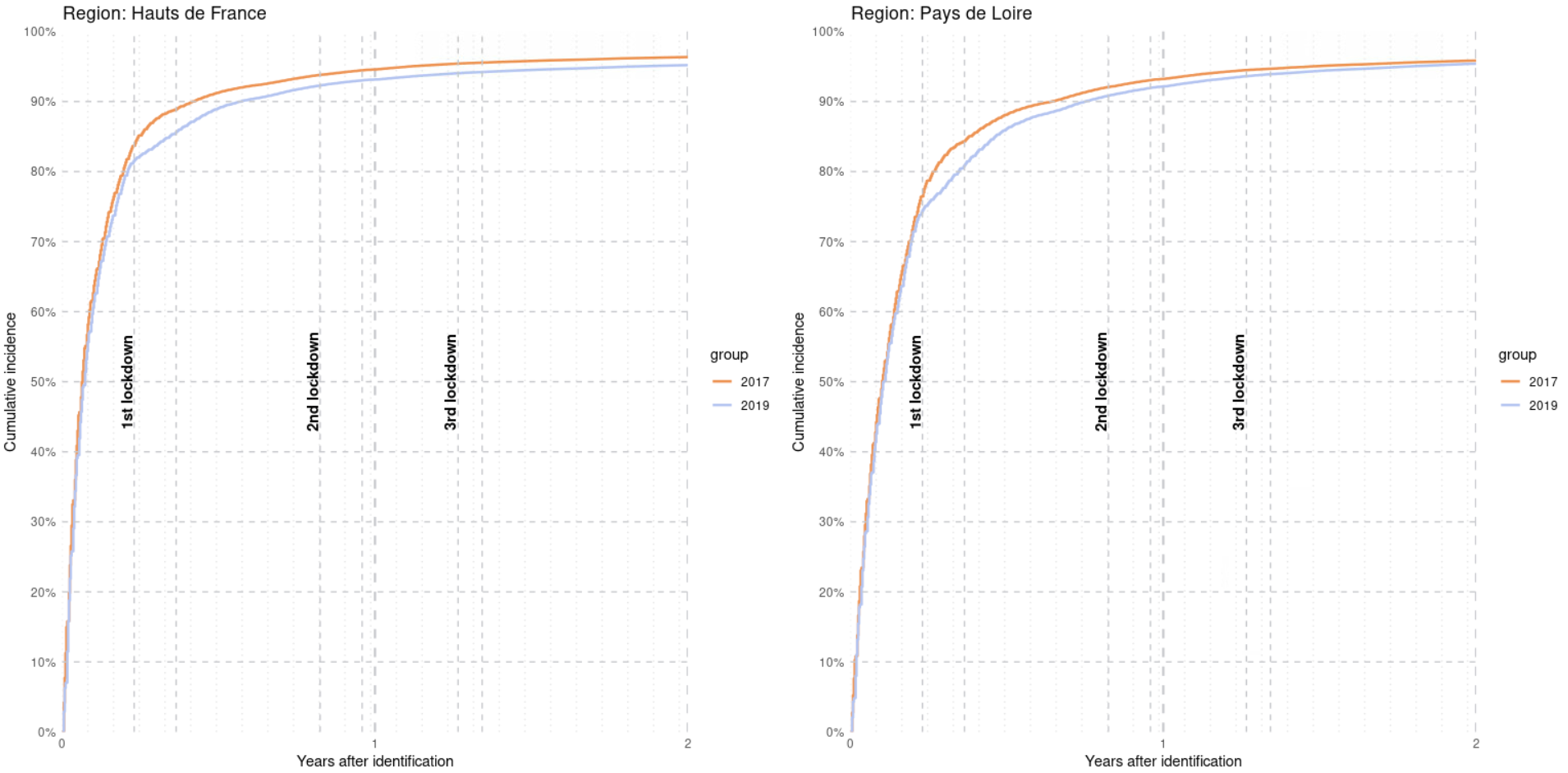


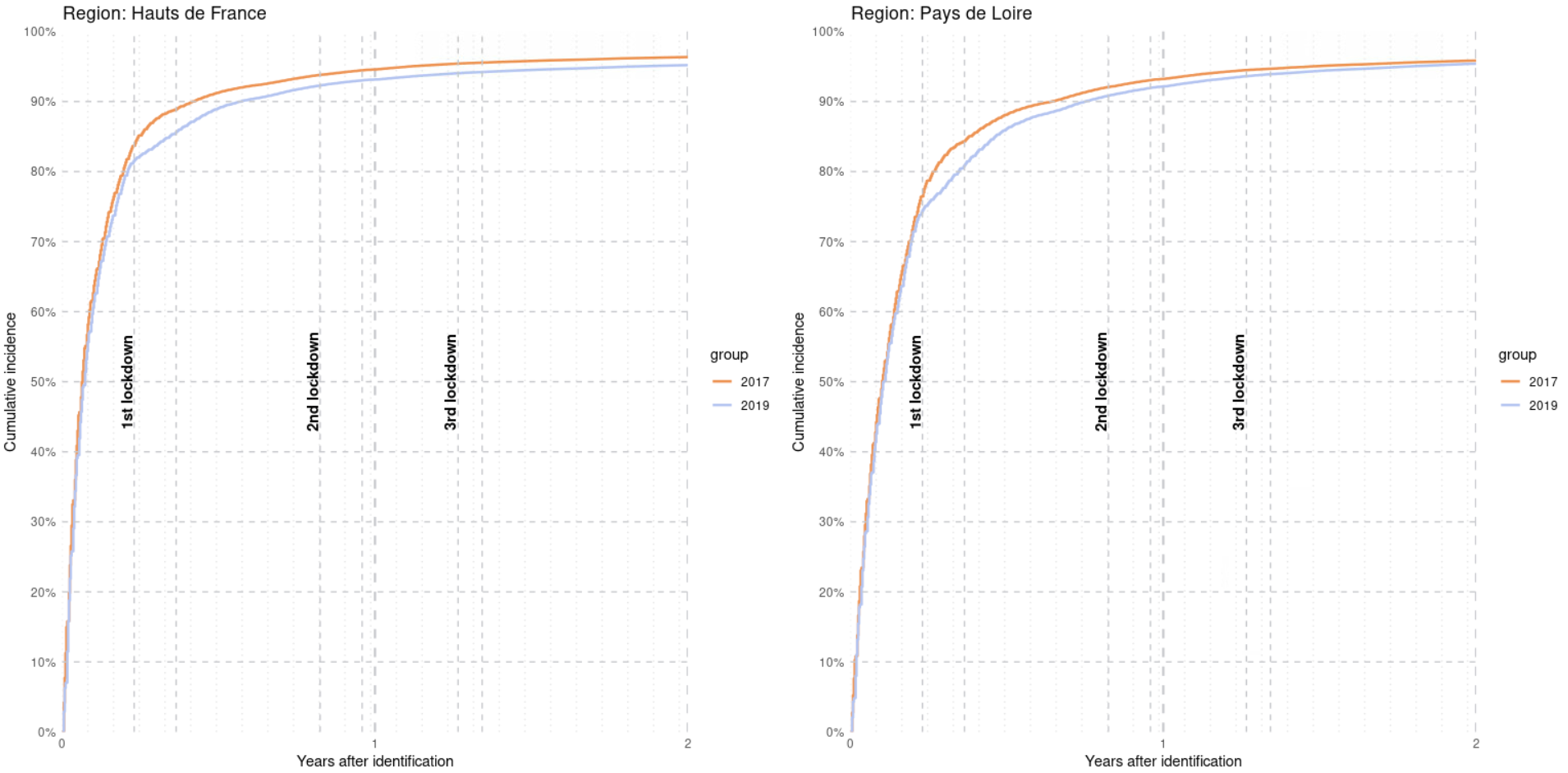


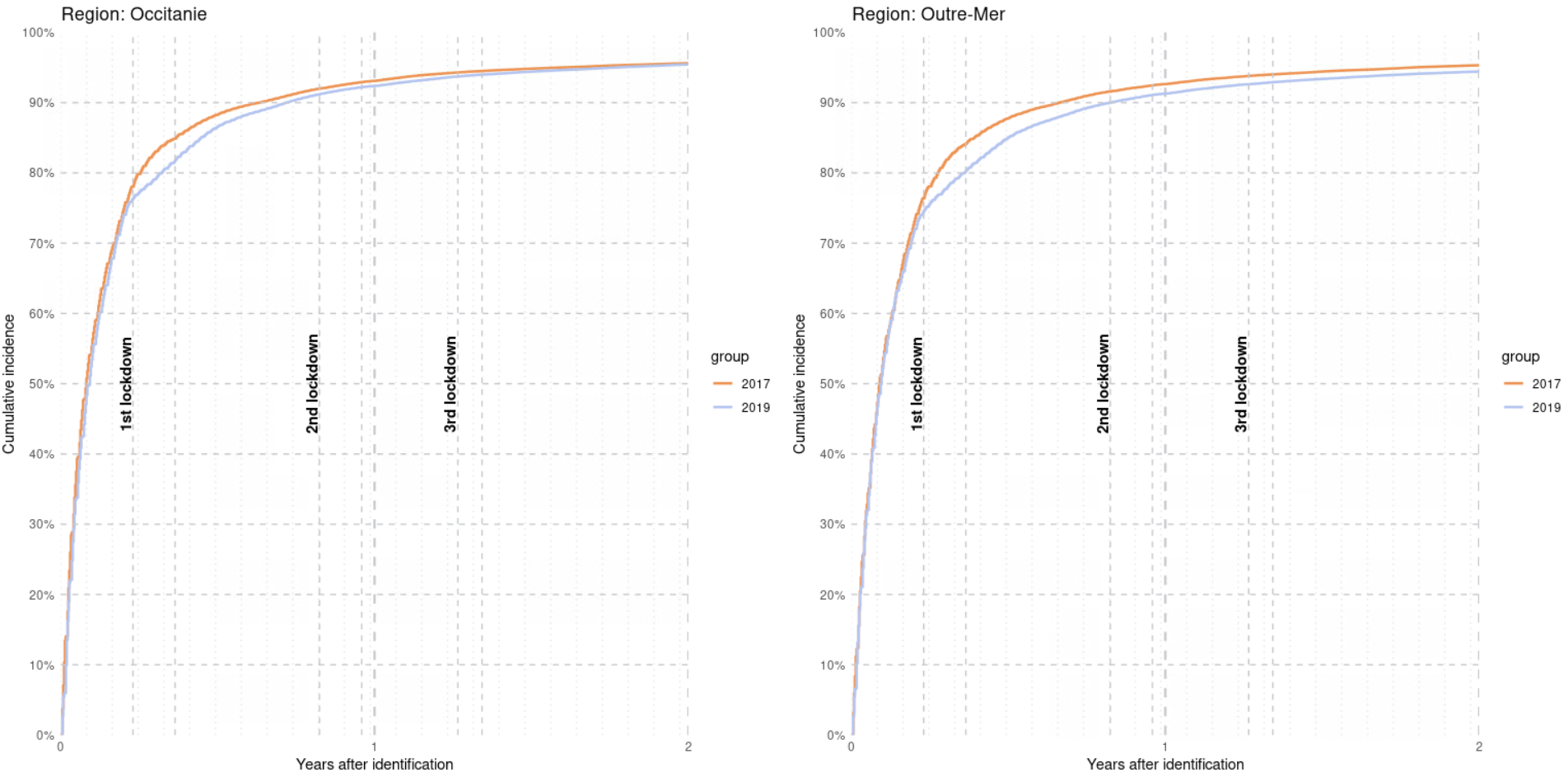


**Figure S7:** Cumulative incidence of a first nephrologist consultation in the 2017 and 2019 cohorts during the follow-up, by region


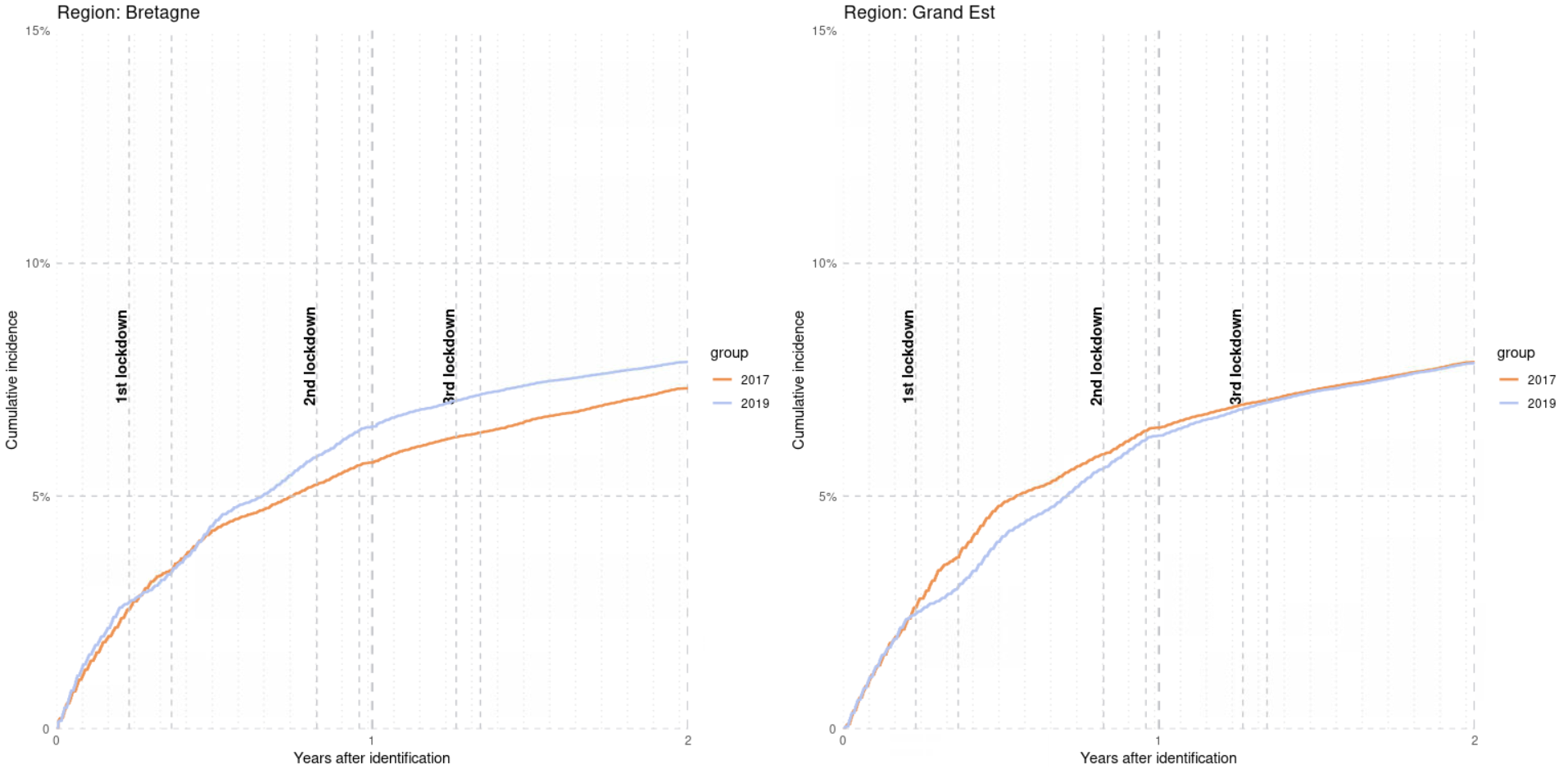


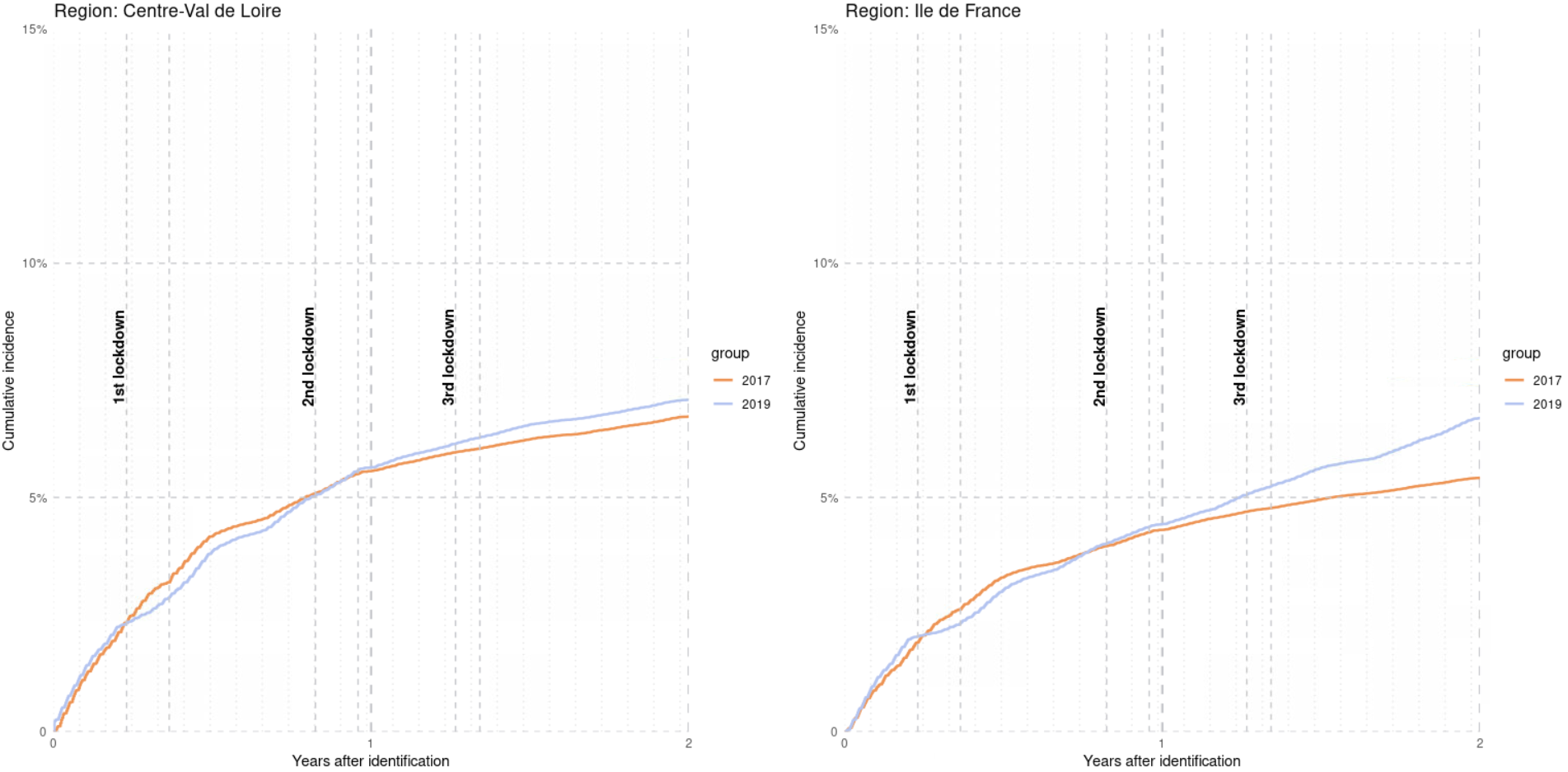


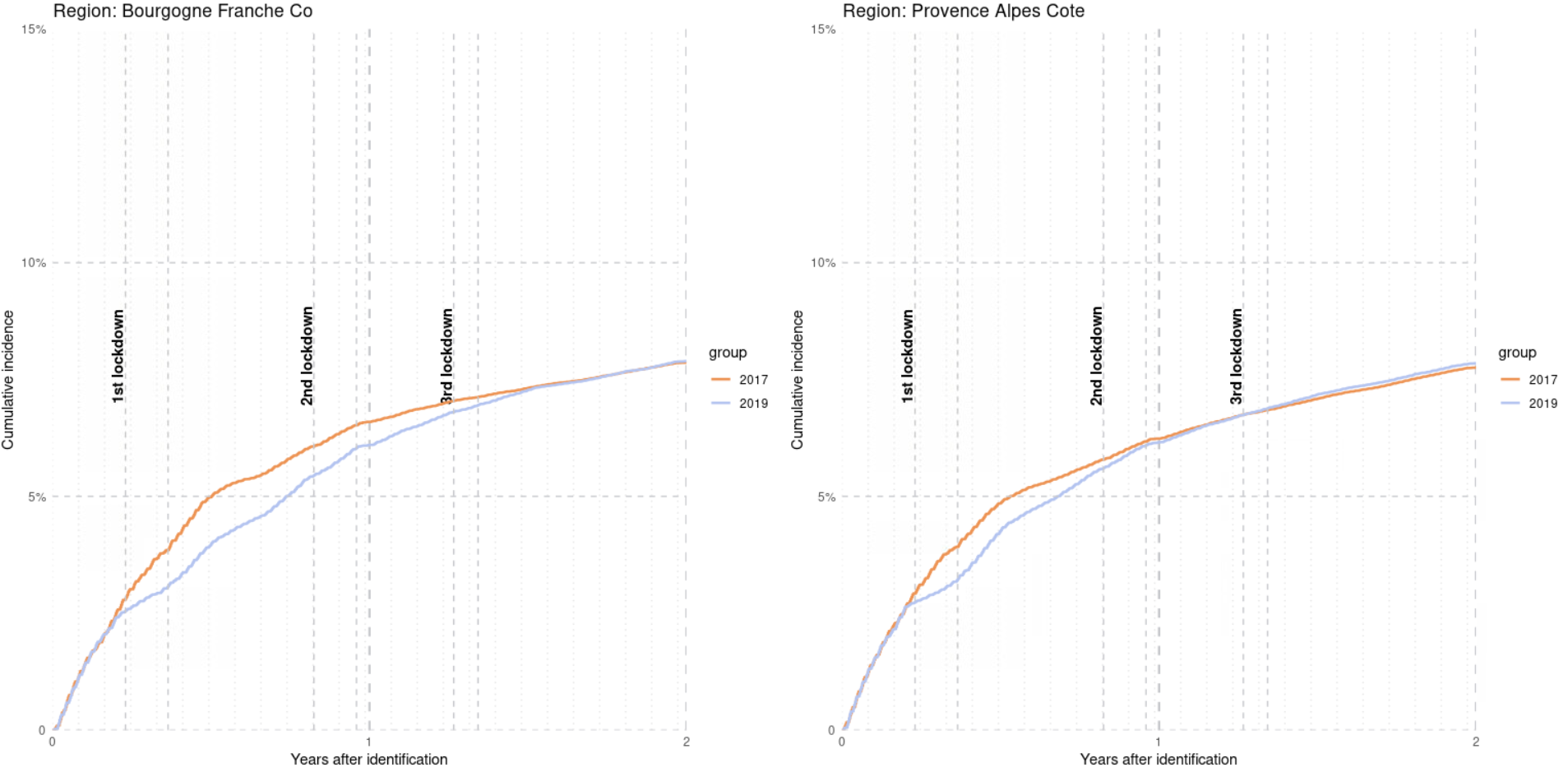


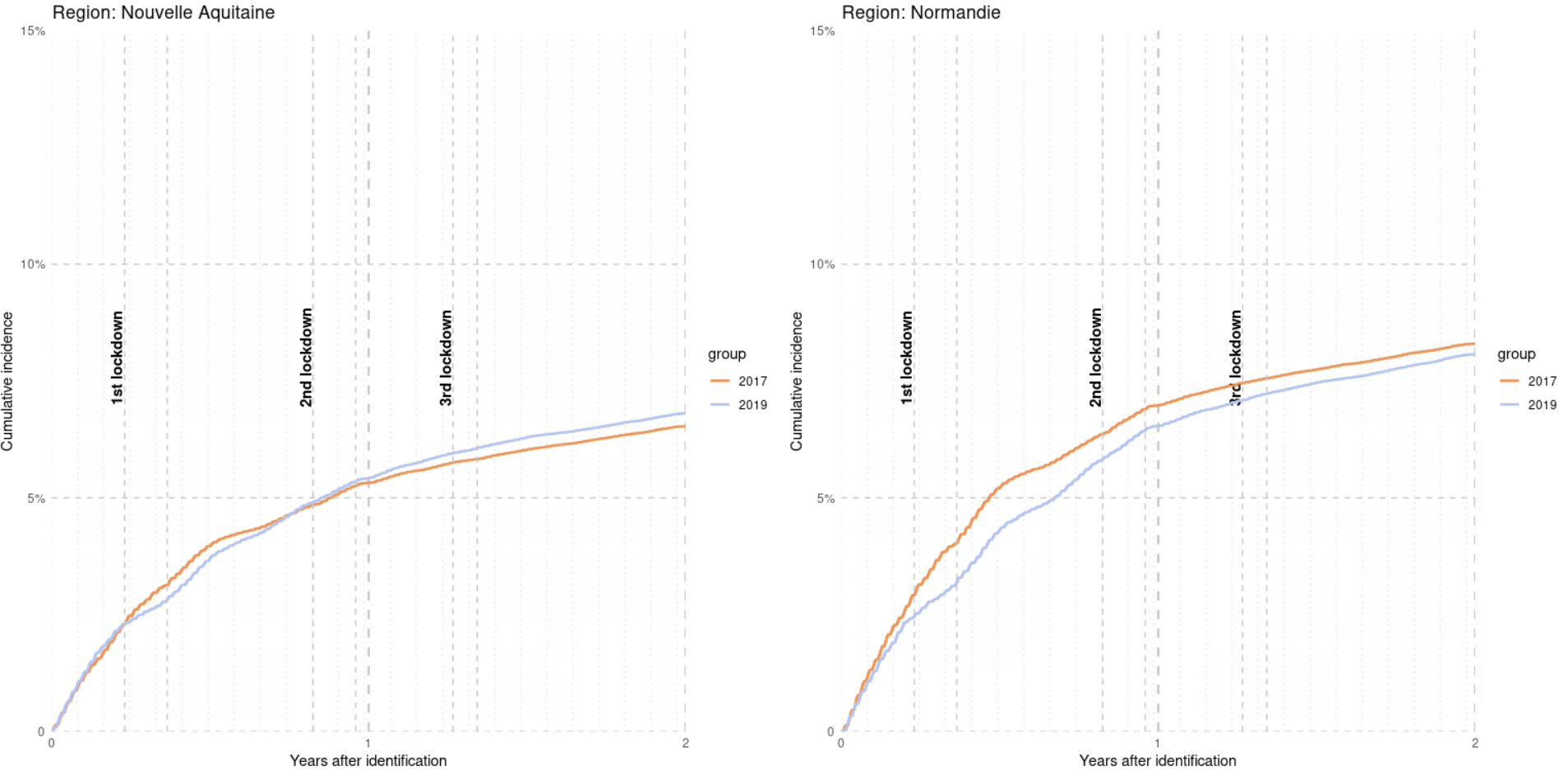


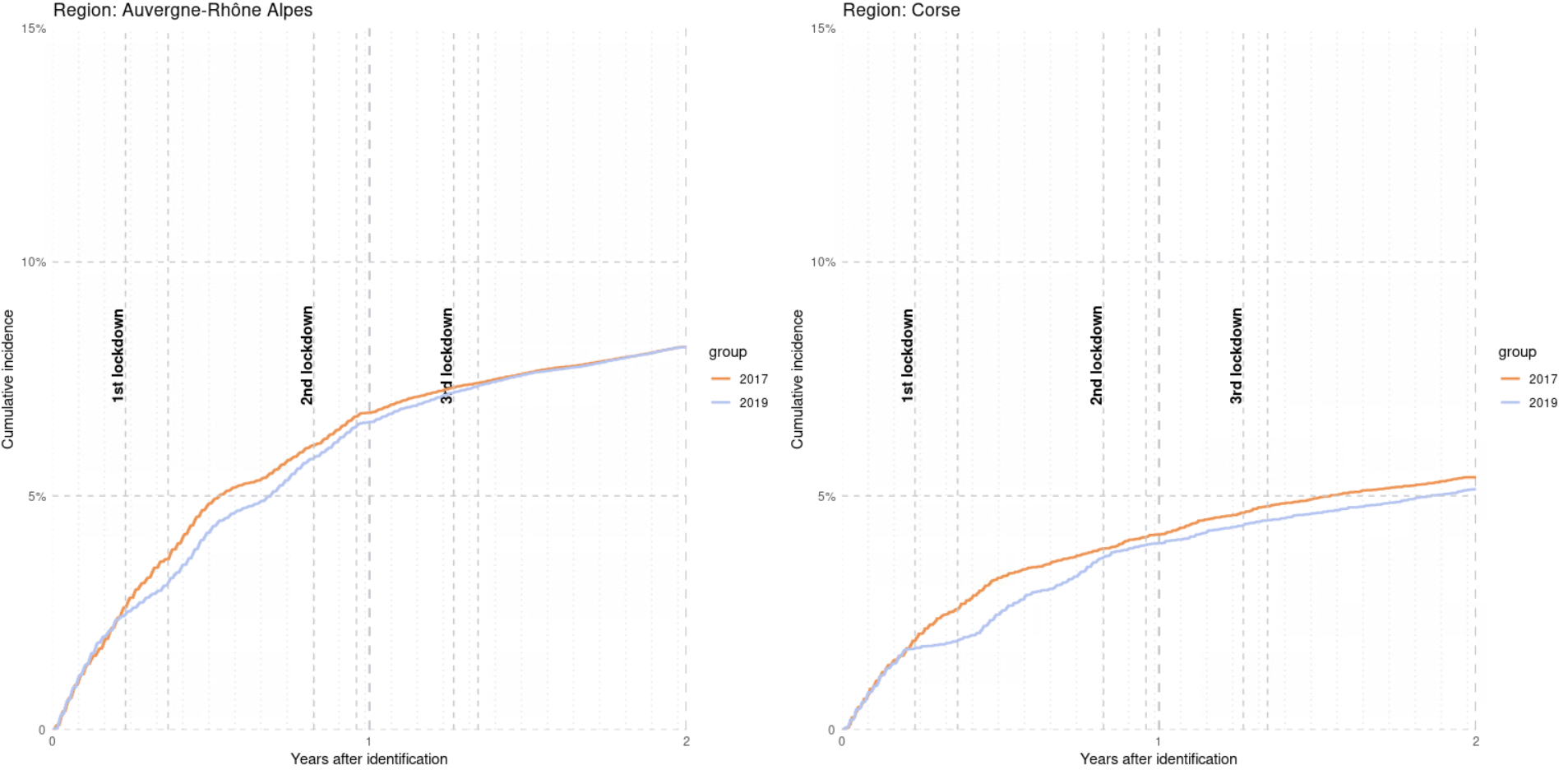


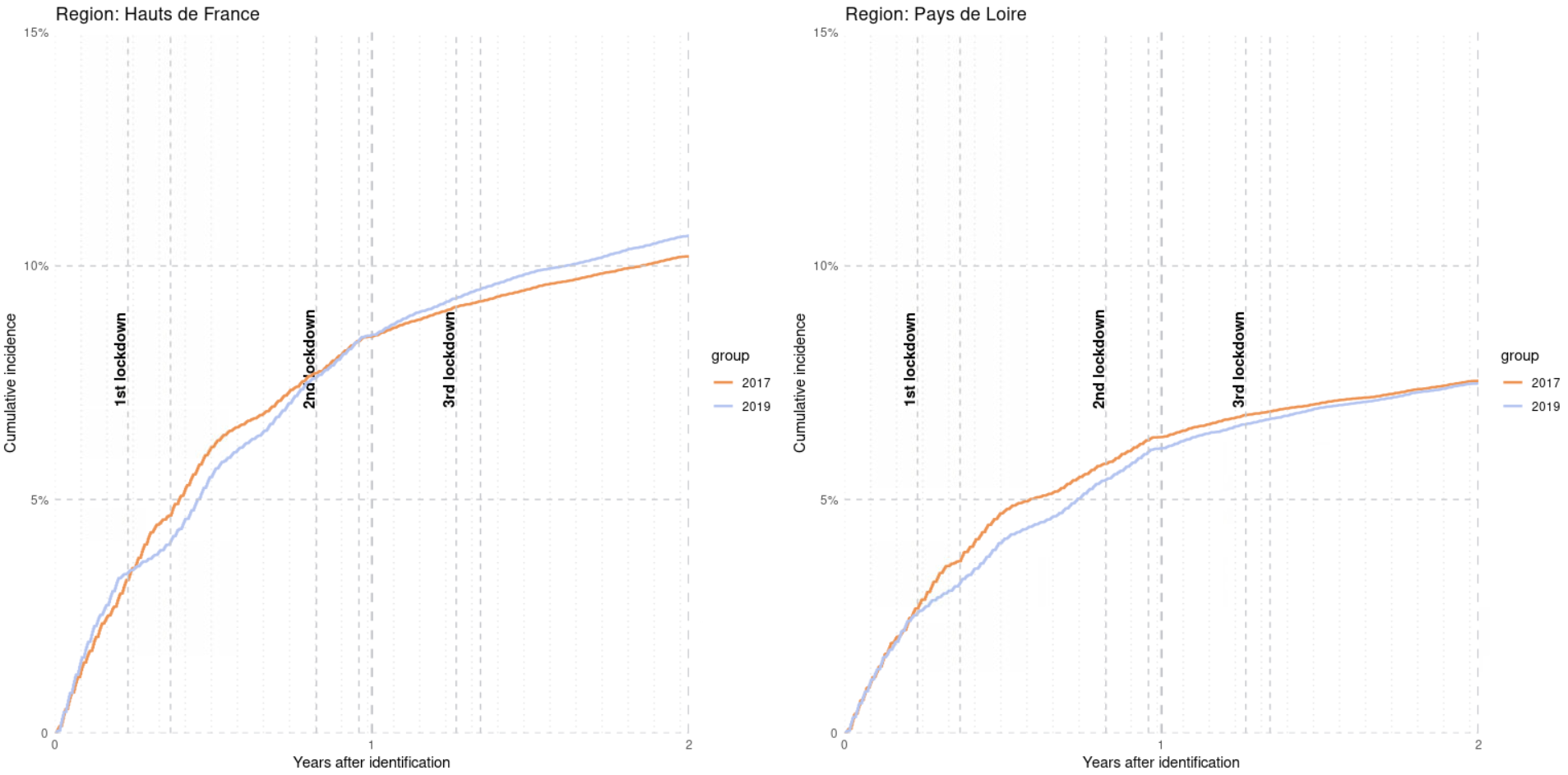

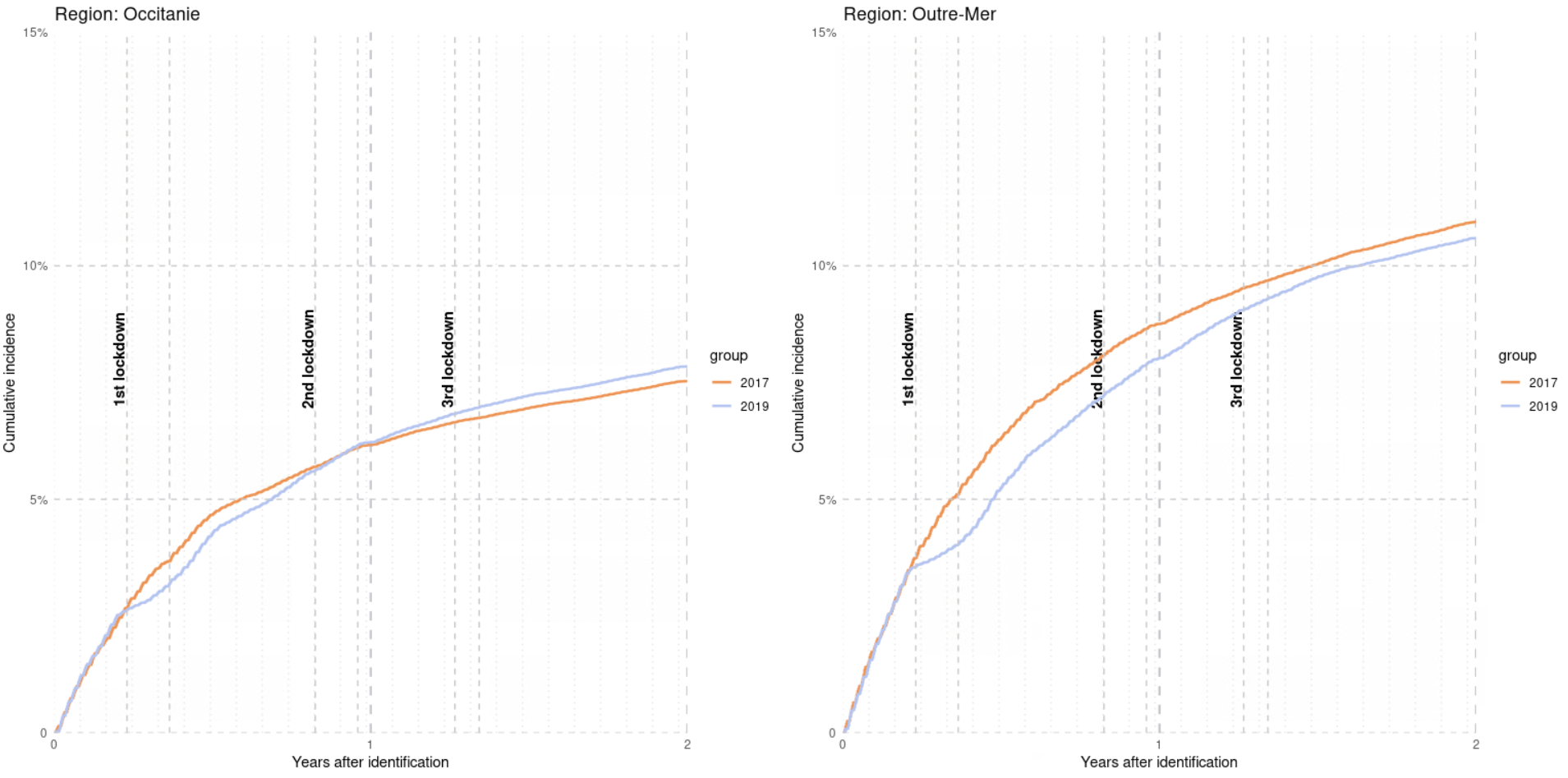

Supplement: Supplementary file 1 — Supplementary file1 (DOCX 6425 KB) [file 40620_2025_2376_MOESM1_ESM.docx]
